# Brain Connectivity Yields Insights into the Pathogenesis of Epilepsy and Subtypes: Evidence from Mendelian Randomization Analysis

**DOI:** 10.34133/hds.0283

**Published:** 2025-08-05

**Authors:** Zhipeng He, Shishi Tang, Yurong Hu, Yuxuan Li, Junhao Liang, Li Fang, Miaoxin Li, Ziyi Chen, Yi Zhou

**Affiliations:** ^1^ Zhongshan School of Medicine, Sun Yat-sen University, Guangzhou, Guangdong, China.; ^2^ School of Computer Science, China University of Geosciences, Wuhan, Hubei, China.; ^3^ The First Affiliated Hospital, Sun Yat-sen University, Guangzhou, Guangdong, China.

## Abstract

**Background:** Alterations of brain connectivity within resting-state networks (RSNs) have been widely reported in observational studies on epilepsy. However, the causal relationship between epilepsy and structural connectivity (SC)/functional connectivity (FC) within RSNs remain unclear. We conducted a bidirectional two-sample Mendelian randomization (MR) to explore the causal relationship between epilepsy subtypes and brain connectivity properties within RSNs. **Methods:** Genetic instruments were obtained from the latest genome-wide association studies (GWAS) of 69,995 individuals (*N*_cases_ = 27,559, *N*_controls_ = 42,436) issued by the International League Against Epilepsy. The GWAS summary SC/FC data within RSNs (*N*_SC_ = 23,985, *N*_FC_ = 24,336) were sourced from the Center for Neurogenomics and Cognitive Research. We investigate the causal relationship between epilepsy subtypes and brain connectivity within RSNs through a bidirectional two-sample MR analysis. **Results:** We found that the increased risk of generalized genetic epilepsy is consistent with a causal effect on dorsal attention and somatomotor FC. In the reverse MR analysis, there was no suggestive causal effect of FC/SC connectivity on epilepsy subtypes. **Conclusions:** This study shed light on the associations of FC/SC levels within the RSNs and epilepsy along with its subtypes. This insight could yield crucial intervention strategies to different subtypes of epilepsy at the level of brain structure and functional networks.

## Introduction

Epilepsy is one of the most common severe central nervous system diseases affecting people of all ages [1]. More than 70 million people worldwide are affected by it, and 80% of these patients live in low- and middle-income countries, which imposes a considerable economic burden [2]. Epilepsy is characterized by sudden and recurrent seizures. It is estimated that, despite continuous medication, 25% to 30% of patients with epilepsy still experience uncontrolled seizures [3]. Therefore, from the perspective of primary prevention, it is crucial to identify the modifiable risk factors for epilepsy.

Epilepsy has long been a focus of neuroscience research. Alterations in functional connectivity (FC) and structural connectivity (SC) are associated with cognitive decline and recovery in patients with epilepsy. In recent years, many studies have employed methods of large-scale brain network, aiming to reveal the relationship between epilepsy and changes in brain functional and structural networks. Coito et al. [4] utilized high-density electroencephalography (EEG) technology to deeply explore the changes in directional FC of patients with temporal lobe epilepsy during the absence of interictal spikes. They found important differences in the connectivity of regions such as the default mode network between patients and healthy controls. Meanwhile, based on EEG source imaging, Coito et al. [5] applied brain FC to patients with temporal lobe epilepsy and analyzed the connectivity differences in different frequency bands. This achievement provides crucial clues for understanding the abnormal brain functions of epilepsy patients. Bartolomei et al. [6] explored the roles of stereo-electroencephalography and signal analysis in defining the epileptic network, which has deepened our understanding of the epileptic network. Obaid et al. [7] sought to assess the SC pattern in patients with operculo insular epilepsy and evaluated whether such changes revealed a characteristic and potentially specific distribution of SC alterations.

However, these existing studies have limitations in assessing the impact of brain connectivity as a risk factor on different epilepsy subtypes. Most of these observational studies focusing on the effects of brain connectivity in epilepsy adopt a cross-sectional design. Cross-sectional studies are not suitable for investigating causal relationships [8]. It remains unclear whether there is a causal relationship between specific epilepsy subtypes and changes in FC and SC within resting-state networks (RSNs). The FC and SC within RSNs is crucial for the cognitive performance of epilepsy patients. To better understand the contribution of spatial correlations in the brain during the resting state, Yeo et al. [9] classified the connectivity between different brain regions in the resting state into seven typical intrinsic connectivity networks (ICNs), which is also commonly known as Yeo’s 7network. It can also be referred to as 7 RNSs. These ICNs are large-scale networks in the brain [10]. They represent the active FC and SC between different brain regions when a person is not performing any specific task. The connectivity is inherent to brain function and are involved in various cognitive processes.

Given the close relationship between FC and SC within RSNs and epilepsy, it is necessary to verify the true causal relationship. Such an association would indicate a potentially modifiable risk of epilepsy or an previously underappreciated consequence of epilepsy. Currently, the ideal way to explore causal relationships is through randomized controlled trials (RCTs), but these trials are time-consuming and may be difficult to implement or unfeasible due to ethical limitations [11]. Mendelian randomization (MR) is another robust and reliable causal inference method that infers the potential causal relationship between exposure and outcome by using genetic variants as instrumental variables (IVs) [12]. Utilizing the causal relationships established in MR analysis can not only provide important candidate targets for future RCT studies, but also offer new insights into the biological mechanisms of the disease. Currently, the genetic predispositions of FC and SC of ICNs related to the risk of epilepsy subtypes using MR have been scarcely studied.

**Fig. 1.**
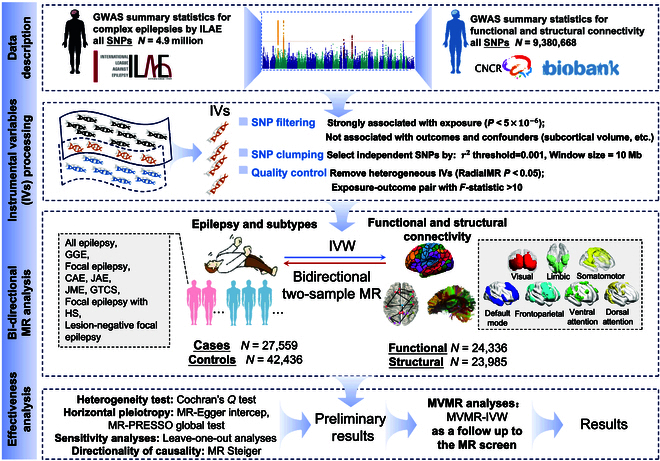
Study flowchart of the causal relationships between epilepsy subtypes and brain functional and structural connectivity by using bidirectional two-sample MR analysis.

Therefore, we conducted a bidirectional two sample MR analysis. The study flowchart is shown in Fig. 1. The forward MR aimed to clarify the impact of epilepsy and its subtypes on the levels of FC or SC within RSNs. The reverse MR, on the other hand, focused on exploring whether changes in the levels of FC or SC in RSNs were risk factors for epilepsy and its subtypes. Through this bidirectional MR, we were able to find which epilepsy subtypes affect which brain network connectivity, and whether changes in the levels of FC or SC in RSNs could potentially be risk factors for epilepsy. This study is expected to provide a perspective for the pathogenesis of epilepsy by MR and also lay the foundation for the development of potential treatment strategies.

## Methods

### Data sources

Genetic variants of phenotypic traits exhibit heterogeneity across different racial or national subgroups. Such heterogeneity can lead to biased effect estimates as the genetic variants used as IVs may not achieve randomization in the presence of confounding factors [13]. To avoid heterogeneity among populations with different genetic backgrounds, we specifically selected individuals of uniform European ancestry as our research subjects when constructing the MR model.

We utilized the recent genome-wide association studies (GWAS) meta-analysis summary statistics of epilepsy [14], which was publicly released by the International League Against Epilepsy (ILAE), encompassing data from 29,000 participants. GWAS summary data from European populations with epilepsy were exclusively chosen. Among them, seizures and epilepsy syndromes were diagnosed according to the ILAE classification. The epilepsy experts at each participating site evaluated all cases and classified them into subtypes based on EEG, imaging, and clinical history. The phenotypic categories of epilepsy included generalized epilepsy, focal epilepsy, and unclassified epilepsy. The genetic seizures with generalized epilepsy were further divided into (a) pediatric absence seizures (childhood absence epilepsy [CAE]), (b) juvenile absence epilepsy (JAE), (c) juvenile myoclonic epilepsy (JME), (d) generalized seizures with tonic–clonic seizures (GTCS) alone, using spiking and wave electroencephalogram, and (e) not as otherwise stated. The subphenotypes of focal epilepsy include (a) focal seizures (focal HS) with HS, (b) lesions other than HS, (c) negative lesions, and (d) not otherwise specified. When exploring the impact of brain SC and FC on epilepsy, we used all epilepsy, generalized epilepsy, focal epilepsy, CAE, JAE, JME, GTCS, focal epilepsy with HS, and lesion-negative focal epilepsy as the outcome variables. Conversely, we analyzed the effects of epilepsy on SC and FC within the RSNs by using these epilepsy types as exposure.

UK Biobank (UKBB) and the Center for Neurogenomics and Cognitive Research (CNCR) revealed the genetic basis of brain function and structural measures [15,16], providing a potential opportunity to clarify the causal relationship of neuroimaging studies in epilepsy. In this study, we analyzed GWAS data on FC/SC within 7 RSNs involving European descent. Resting-state functional magnetic resonance imaging (rsfMRI) and diffusion-weighted imaging for brain imaging in UKBB as well as T1 surface models and structural segmentation files of FreeSurfer were used to construct brain FC and SC within 7 RSNs. The extraction of RSNs is accomplished by CNCR [16]. When computing the FC and SC, the Cammoun subparcellations of the Desikan–Killiany atlas were used, which divides the brain surface into different cortical regions. For the calculation of FC, it is obtained by measuring the average correlation of brain region activation signals over time within each RSN; for the measurement of SC, it is calculated by the average fractional anisotropy (FA) of the weight for the reconstruction of fibers.

We selected the GWAS information on FC and SC corresponding to ICNs, including the default mode network, ventral attention network, dorsal attention network, visual network, limbic network, somatomotor network, and frontoparietal network. To assess the genetic correlation between FC/SC within RSN and epilepsy, considering whether the onset of epilepsy and subtypes are affected by global FC/SC or whether epilepsy alters the overall brain connectivity, we also included the GWAS of global FC and global SC phenotypes in this study. Details of these data can be found in the original publications and are shown in Table.

**Table.** Details of the data sources used in this study

Epilepsy phenotype	Phenotype description	Sample size
Genetic generalized epilepsy (GGE)	Generalized epilepsy, not otherwise specified, with spike and wave EEG	3,024 (4.320%)
	Childhood absence epilepsy (CAE)	1,049 (1.499%)
	Juvenile absence epilepsy (JAE)	662 (0.945%)
	Juvenile myoclonic epilepsy (JME)	1,732 (2.474%)
	GTCS only, with spike and wave EEG	485 (0.692%)
Focal epilepsy	Focal epilepsy, not otherwise specified	3,688 (5.268%)
	Focal epilepsy, documented lesion negative	5,778 (8.254%)
	Focal epilepsy, documented hippocampal sclerosis (HS)	1,260 (1.800%)
	Focal epilepsy, documented lesion other than HS	4,213 (6.019%)
Unclassified	Epilepsy, not otherwise specified	5,668 (8.097%)
	Cases	27,559 (39.37%)
	Controls	42,436 (60.63%)
	Total subjects	69,995
Connectivity phenotype	Phenotype description	Sample size
Functional connectivity (FC)	FC within resting-state network, including dorsal attention, frontoparietal, limbic, somatomotor, ventral attention, visual, default mode, and global networks	24,336 (50.363%)
Structural connectivity (SC)	SC within resting-state network, including dorsal attention, frontoparietal, limbic, somatomotor, ventral attention, visual, default mode, and global networks	23,985 (49.636%)
	Total subjects	48,321

### Instrument selection and quality control

**Fig. 2.**
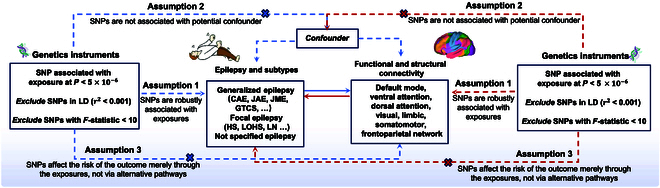
Analysis framework for the three key hypotheses of MR.

The IVs in the MR study must meet the three core assumptions of association, independence, and exclusivity [17–19] (Fig. 2): (a) IVs are robustly associated with exposures; (b) IVs are not associated with potential confounders; and (c) IVs affect the risk of the outcome merely through the exposures, not via alternative pathways.

In order to address the strong correlation between IVs and exposure factors, significant single-nucleotide polymorphisms (SNPs) were selected at a genome-wide dominant threshold of *P* < 5 × $10^{-6}$ for FC and SC (Table S1). Relevant SNPs were utilized as candidate IVs for the following bidirectional MR analysis. Similarly, for epilepsy and subtypes, a threshold of *P* < 5 × $10^{-6}$ was employed (Table S2). As there were sufficient available IVs for MR analysis at this threshold and some related MR studies [20,21] also used *P* < 5 × $10^{-6}$as the threshold, an appropriate threshold up to $10^{-6}$ was chosen.

To ensure that the candidate IVs meet the independence assumption, we use a rigorous clustering procedure to remove the SNPs in linkage disequilibrium (LD). Specifically, utilizing the TwoSampleMR [22] in R package, the parameter was set to $r^{2}$ = 0.001 and *kb* = 10,000 to remove the SNP with $r^{2}$ above 0.001 within 10 MB to exclude the effect of LD. The LDtrait tool [23] (https://ldlink.nih.gov) was used to verify whether the selected SNP was also associated with other phenotypes. Potential confounders include subcortical volume, white matter microstructure, and self-reported educational attainment (Table S9).

The effectiveness of the selected SNPs was evaluated by calculating the *F*-statistic, with *F* > 10 serving as an indicator of robust instruments. The calculation formula of the *F*-statistic is:

$$F=R^{2}\left(N-k-1\right)∕k\left(1-R^{2}\right)$$ (1)

$$R^{2}=\frac{2{\hat{\beta }}^{2}\mathrm{MAF}\left(1-\mathrm{MAF}\right)}{2{\hat{\beta }}^{2}\mathrm{MAF}\left(1-\mathrm{MAF}\right)+{\left(\mathrm{se}\left(\hat{\beta }\right)\right)}^{2}2\text{NMAF}\left(1-\mathrm{MAF}\right)}$$ (2)

where $R^{2}$ represents the proportion of phenotypic variance explained by genetic variation, used to assess the contribution of the SNP to variation in a particular trait. *β* is the effect size, *MAF* refers to the minor allele frequency, *N* represents the sample size, and *k* is the number of IVs. *F*-statistics were considered to have sufficient explanatory power for phenotypic variation. *F*-statistics indicated that all genetic instruments were suitable for MR analysis (see Table S3).

### Bidirectional two-sample MR analyses

We performed a bidirectional two-sample MR analysis to investigate the causal relationship between epilepsy subtypes and the SC/FC within RSNs. For forward MR analysis, epilepsy and subtypes were used as exposures, while brain SC and FC were considered as outcomes. Conversely, in the reverse MR analysis, brain SC and FC were utilized as exposures, with epilepsy and subtypes as the outcomes.

The inverse variance weighting (IVW) was selected as the preferred approach to ascertain the causal effect of exposure on outcome. IVW has the highest statistical power, but the IVW method only gives unbiased estimates when IVs are effective or without pleiotropy [24]. Therefore, we used four other MR methods as a complement to detect the robustness and reliability of the results, including the weighted median [25], MR Egger [26], simple mode [27], and weighted mode [28]. To ensure the robustness of MR results, the same direction of five MR analysis methods is considered as a meaningful causal relationship, and at least 5 SNPs can be used for IVs.

### Sensitivity analyses

We performed a series of sensitivity analyses to examine the robustness of the MR results. We conducted the leave-one-out analysis (i.e., removing one IV at a time and re-estimating the causal relationship) to test whether the causal relationship was driven by a specific SNP. The Cochran’s *Q* test in IVW is used to evaluate whether the effect estimates of different IVs are consistent. When the *P* value of Cochran’s *Q* test is less than 0.05, it indicates significant differences between the IVs, suggesting potential heterogeneity.

We calculated the intercept of the MR-Egger regression to evaluate whether genetic variants exhibit pleiotropy. If the intercept of the MR-Egger regression is significantly different from zero, it indicates the existence of horizontal pleiotropy (*P* < 0.05) [29]. Furthermore, the presence of pleiotropy was also assessed using the MR-PRESSO test. To assess causality using weighted median regression, at least 50% of the information should come from nonpleiotropic SNPs to obtain an unbiased estimate. We further conducted the MR-Steiger test to estimate the potential reverse causal effects. In addition, we conducted multivariable MR (MVMR) analyses as a follow-up validation to the two-sample MR screening.

## Results

### Effects of epilepsy and subtypes on specific brain SC

In the preliminary forward MR analysis, increased risk of CAE caused increased connectivity in the ventral attention network. Meanwhile, high genetic susceptibility to focal epilepsy will enhance the connectivity of the global brain structural network. However, considering the confounder of generalized genetic epilepsy (GGE), there was no suggestive evidence of the effect of CAE on the ventral attention network ($P_{\text{MVMR}-\mathrm{IVW}}$ = 0.25567). Considering the confounder of self-reported educational attainment, the effect of focal epilepsy on the global network was not significant ($P_{\text{MVMR}-\mathrm{IVW}}$ = 0.05332).

### Effects of epilepsy and subtypes on specific brain FC

**Fig. 3.**
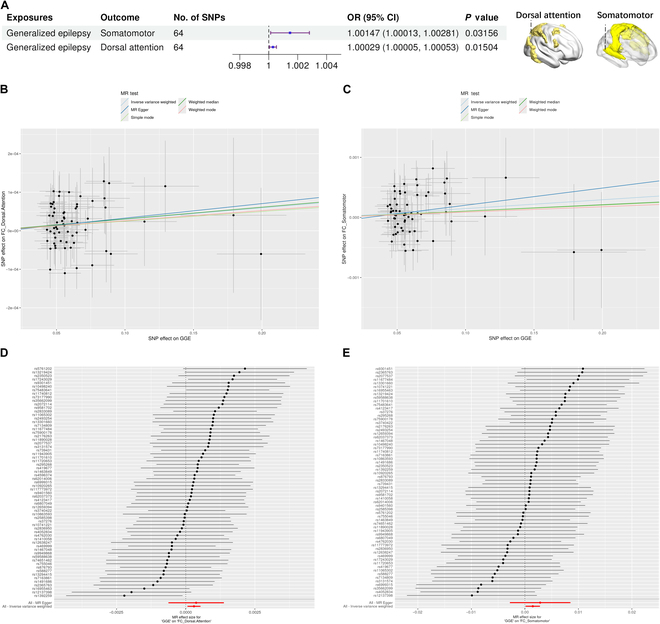
The causal effect of epilepsy on brain functional connectivity (FC) estimated by the MR. (A) Summary MR estimates for the effect of epilepsy on brain FC with *P-ivw* < 0.05. (B) Scatterplot of SNP associated with the GGE versus FC of dorsal attention network, with the slope of each line corresponding to the estimated MR effect per method. (C) Scatterplot of SNP associated with GGE versus FC of the somatomotor network. (D) Forest plot of individual and combined SNP MR-estimated effect sizes for GGE on FC of the dorsal attention network. (E) Forest plot of individual and combined SNP MR-estimated effect sizes for GGE on FC of the somatomotor network.

GGE had a suggestive effect on the FC changes of the dorsal attention network, somatomotor network, and limbic network (see Table S4). These are the core ICNs that support cognitive functions such as spatial perception and memory. We observed a suggestive correlation between GGE and the FC of these ICNs (Fig. 3 and see Figs. S1 and S4). In the multivariable MR validation analysis, even after considering the confounder of self-reported educational attainment, there was still suggestive evidence of the effect of GGE on the dorsal attention and the somatomotor network ($P_{\text{MVMR}-\mathrm{IVW}}$ < 0.05), but the effect of GGE on the limbic network was not significant ($P_{\text{MVMR}-\mathrm{IVW}}$ = 0.054). For focal epilepsy, we found that its genetic susceptibility with dorsal attention network FC decreased was associated. Focal lesion negative was suggestively associated with the decreased frontoparietal network FC. After considering the confounder of self-reported educational attainment, the effect of focal epilepsy on the limbic network was not significant ($P_{\text{MVMR}-\mathrm{IVW}}$ = 0.054).

The above causal relationships did not have the reverse causal effect as tested by the MR-Steiger test (see Table S8). The results of the IVW method for the above causal relationships were consistent with the direction of results assessed by other supplementary MR methods (see Table S4). Both Cochran’s *Q* test for heterogeneity and MR-Egger regression analysis for pleiotropy indicated that there was no heterogeneity and no horizontal pleiotropy in genetic variation (see Table S6). Further, the MR-PRESSO test showed no horizontal pleiotropy (see Table S7). Leave-one-out analysis also indicated that the causal effect was not driven by any specific instrumental SNP (see Fig. S3).

### Effects of specific brain SC on epilepsy and subtypes

To further explore the causal effects of specific brain SC on epilepsy and its subtypes, we performed the reverse MR analysis. Note that we conducted SNPs lookup in LDTrait. SNPs rs2003585 and rs2267161 were associated with subcortical volume and white matter microstructure. After removing SNPs associated with confounders, no significant results of the effect of brain SC on epilepsy were found.

### Effects of specific brain FC on epilepsy and subtypes

Using the information of genetic variants associated with FC, visual networks and somatomotor networks had significant impacts on all epilepsy, and the five MR analyses demonstrated consistent directional causal effects (see Table S5). However, the number of instrumental SNPs in the visual and somatomotor networks was less than 5; therefore, it was not possible to perform a sensitivity analysis and ensure the robustness of the conclusion.

## Discussion

The concept of epilepsy as a network disease has opened up a new perspective for exploring the diagnosis and treatment strategies of epilepsy and subtypes [30–32]. The SC and FC within RSNs show significant changes in epilepsy and its different subtypes. Diffusion tensor imaging (DTI) and fMRI provide important means for studying the SC and FC changes of brain networks in epilepsy patients [33,34]. Larivière et al. [35] conducted a worldwide Enhancing Neuro Imaging Genetics through Meta-Analysis (ENIGMA) study and found that structural network alterations in epilepsy followed the axes of epilepsy risk gene expression. Their research focused on the relationship between structural networks and gene expression patterns in epilepsy, revealing that topological changes in brain networks were related to the expression of risk genes for the epilepsy. This is complementary to our study, as we focused on exploring the causal relationship between epilepsy and brain connectivity through MR analysis. Together, these findings deepen our understanding of the pathogenesis of epilepsy.

GGE, a common type of epilepsy with genetic inheritance, is closely related to abnormal activities in the ICNs. This abnormal activity can lead to cognitive, attention dysfunction, and emotional regulation problems [36,37]. This impact may originate from abnormalities in the neural pathways between multiple brain regions [38]. Our MR analysis reveals converging evidence for epilepsy as a brain network disorder, bridging genetic predisposition with macroscale network dysfunction. The results of MR demonstrate suggestive associations between generalized epilepsy and functional alterations in somatomotor networks (OR = 1.00147, 95% CI 1.00013 to 1.00281, *P* = 0.03156) and dorsal attention networks (OR = 1.00029, 95% CI 1.00005 to 1.00053, *P* = 0.01504). While the effect sizes appear modest, their directional consistency with neuroimaging findings strengthens the biological plausibility.

These associations may reflect polygenic influences on network stability mechanisms described by Courtiol et al. [39], where subtle shifts in dynamic metastability could amplify genetic risk into macroscopic network alterations. Notably, the affected networks align with Xie et al.’s observation [40] of sensory-transmodal differentiation loss in temporal lobe epilepsy, suggesting shared pathomechanisms across epilepsy subtypes. The structural basis of these functional–genetic correlations may involve the cortical thickness covariance patterns identified by Duma et al. [41]. Their finding of structure–function coupling in limbic regions provides a potential substrate for how genetic variants influencing cortical development could predispose to network instability.

Specifically, the dorsal attention network alterations in our analysis may relate to the network disintegration reported by Caciagli et al. [42], as these systems exhibit competitive interactions in healthy cognition. The simultaneous involvement of somatomotor networks echoes recent work on ictal propagation pathways, where SC between motor and epileptogenic zones facilitates seizure spread.

Methodologically, the MR-derived suggestive evidence may address a limitation in prior network studies—the difficulty in establishing causal directionality. Our findings complement Courtiol et al.’s [39] computational models by providing suggestive evidence that genetic risk factors may tip the balance between global stability and local excitability. The slightly suggestive association with somatomotor networks (wider CI notwithstanding) could reflect the privileged SC of motor areas, as demonstrated in Duma et al.’s [41] wave propagation analysis.

Note that this study used a relatively lenient threshold (*P* < 5.0 × 10^−6^) to screen for SNPs associated with SC/FC, identifying over 3,000 genetic variants. This threshold was chosen to balance statistical power under polygenic backgrounds with the risk of false positives, consistent with strategies employed in recent MR studies of neuroimaging phenotypes [20,21]. Although the large number of SNPs may raise concerns about weak instrument bias or pleiotropy, all IVs exhibited *F*-statistics > 10 (Table S3), and sensitivity analyses (e.g., weighted median method, MR-Egger) showed consistent directional causal effects across approaches (Table S4), validating the robustness of the results. By excluding SNPs associated with confounders (e.g., educational attainment and subcortical volume) using the LDtrait tool (Table S9) and correcting for potential pleiotropy via MVMR (Table S10), the nonsignificant intercept in MR-Egger regression (*P* > 0.05) and outlier removal in MR-PRESSO analysis (Tables S6 and S7) further indicated that primary findings were not substantially confounded by pleiotropic effects. The polygenic association between SC/FC and epilepsy subtypes suggests that network dysfunction in epilepsy may arise from the cumulative effects of minor-effect genetic variants.

There are several limitations in this study. Firstly, it only considered intra-network correlations and did not consider inter-network correlations. In the future, we will continue to consider the whole-genome genetic correlations between networks. Secondly, as this study only used GWAS data from European populations, it cannot well represent other races and ethnic groups in the world. Thirdly, regarding the gene–environment equivalence assumption, although genetic variations often have a similar impact on exposure as environmental factors do, genetic variations are unlikely to fully mimic environmental changes [43]. This may lead to some bias in our interpretation of the results.

Moreover, we used LDTrait to screen for SNPs associated with confounding factors and employed multiple sensitivity analyses such as the MR-Egger and MR-PRESSO tests to assess pleiotropy. Additionally, we conducted MVMR analyses as a follow-up validation to the two-sample MR screening. Although the current results show no significant horizontal pleiotropy, the potential pleiotropic effects of SNPs cannot be completely ignored. The SNPs might be associated with other biological processes or traits that were not fully explored in this study. This could potentially confound the causal relationships we are investigating between network connectivity and epilepsy. Future studies should further explore these potential pleiotropic associations to gain a more comprehensive understanding of the underlying mechanisms.

In future research, multimodal imaging data could be combined with genetic information, and animal experimental verification techniques such as gene knockout studies will be employed to elucidate the mechanisms underlying altered brain network connectivity in different epilepsy subtypes. Furthermore, future research could integrate single-cell epigenomics and dynamic network models to dissect their synergistic regulatory mechanisms [35,40]. This comprehensive approach can enhance our understanding of the clinical implications of epilepsy and ultimately advance diagnosis and treatment options for patients with epilepsy. It is necessary to strengthen efforts to screen for FC and SC levels within the RSNs during seizure episodes, elucidate their clinical relevance, and investigate their potential role as modifiable risk factors.

## Conclusion

This is a comprehensive MR analysis that reveals the associations between epilepsy subtypes and SC/FC within RSNs. For the effects of epilepsy and subtype on specific FC, we provided suggestive evidence that the dorsal attention and somatomotor network FC is associated with higher GGE risk. In the other directions, although there were some significant results, after removing confounders or performing the MVMR analysis, we observed no significant results in MR estimates. These findings may potentially offer insights into epilepsy prevention strategies and intervention measures targeted at SC and FC within RSNs. To figure out the mechanisms of the association between epilepsy and SC and FC within RSNs, further investigation into the biological functions of these brain regions is needed.

## Data Availability

The data that support the findings of this study are openly available at https://figshare.com/s/fc22c4a8a49ae9b9997c. Summary data from each consortium are obtained from their respective public data repositories. The GWAS summary data that support the findings of this study for epilepsy analyses are publicly available at https://www.epigad.org/. The connectivity properties can be accessed at https://www.fmrib.ox.ac.uk/ukbiobank/gwas_resources/. The GWAS data of FC/SC connectivity within cerebral RSNs data can be obtained from the CNCR committee (https://ctg.cncr.nl/software/summary_statistics/). The GWAS summary statistics used in the MR analysis are shown in Tables S1 and S2. Additional descriptions of these GWAS are provided in the Supplementary Materials. All data for this study were obtained from publicly available GWAS summary statistics, with written informed consent and ethical approval, and ethical approval can be found in the original study.
