# Supplementary material for: Brain Connectivity Yields Insights into the Pathogenesis of Epilepsy and Subtypes: Evidence from Mendelian Randomization Analysis
Source: Health Data Sci. 2025 Aug 5;5:0283. doi: 10.34133/hds.0283 (PMC12324163; doi:10.34133/hds.0283)

**Supplementary information to:**

**Brain connectivity yield insights into the pathogenesis of epilepsy and subtypes: evidence from mendelian randomization analysis**

Zhipeng He Ph.D.1, Shishi Tang Ph.D.1, Yurong Hu Ph.D.2, Yuxuan Li Ph.D.1, Junhao Liang Ph.D.1, Li Fang Ph.D.1, Miaoxin Li Ph.D.1, Ziyi Chen M.D.3*, Yi Zhou M.D., Ph.D.1 *

1. Zhongshan School of Medicine, Sun Yat-sen University, Guangzhou, Guangdong, China.

2. School of computer science, China University of Geosciences, Beijing, China.

3. First Affiliated Hospital, Sun Yat-sen University, Guangzhou, Guangdong, China.

***Address correspondence to:**

1. Yi Zhou, MD, PhD, Department of Medical Informatics, Zhongshan School of Medicine, Sun Yat-sen University, Guangzhou, Guangdong 510080, China. Email: zhouyi@mail.sysu.edu.cn.

2. Ziyi Chen, MD, Department of Neurology, First Affiliated Hospital, Sun Yat-sen University, Guangzhou, Guangdong 510080, China. Email: chenziyi@mail.sysu.edu.cn.

**A list of Supporting Information**

**Supplementary Table 1.** GWAS summary data of functional and structural connectivity at P-value<5.0×${10}^{-6}$.

**Supplementary Table 2.** GWAS summary data of epilepsy and subtypes at P-value<5.0×${10}^{-6}$.

**Supplementary Table 3.** The $R^{2}$ and F-statistics for the genetic instruments and the power for MR.

**Supplementary Table 4.** The results for forward MR estimate.

**Supplementary Table 5.** The results for reverse MR estimate.

**Supplementary Table 6.** Sensitivity analysis of instrumental variables used in MR analyses.

**Supplementary Table 7.** The result of MR-PRESSO test

**Supplementary Table 8.** The result of MR Steiger test

**Supplementary Table 9.** Annotated SNP list by LDTrait tool

**Supplementary Table 10.** Results of the MVMR analysis for two sample MR screening

**Supplementary Table 11.** Results of the FUMA analysis for functional annotation

**Supplementary Figure 1.** The forest plots of the association between genetic predicted epilepsy and subtypes on functional connectivity in MR analysis.

**Supplementary Figure 2.** The funnel plots of the association between genetic predicted epilepsy and subtypes on functional connectivity in MR analysis.

**Supplementary Figure 3.** The leave-one-out analysis of the association between genetic predicted epilepsy and subtypes on functional connectivity in MR analysis.

**Supplementary Figure 4.** The scatter plots of the association between genetic predicted epilepsy and subtypes on functional connectivity in MR analysis.

**Supplementary Figure 5.** The forest plots of the association between genetic predicted epilepsy and subtypes on structural connectivity in MR analysis.

**Supplementary Figure 6.** The funnel plots of the association between genetic predicted epilepsy and subtypes on structural connectivity in MR analysis.

**Supplementary Figure 7.** The leave-one-out analysis of the association between genetic predicted epilepsy and subtypes on structural connectivity in MR analysis.

**Supplementary Figure 8.** The scatter plots of the association between genetic predicted epilepsy and subtypes on structural connectivity in MR analysis.

**Supplementary Figure 9.** The forest plots of the association between genetic predicted structural connectivity on epilepsy and subtypes in MR analysis.

**Supplementary Figure 10.** The funnel plots of the association between genetic predicted structural connectivity on epilepsy and subtypes in MR analysis.

**Supplementary Figure 11.** The leave-one-out analysis of the association between genetic predicted structural connectivity on epilepsy and subtypes in MR analysis.

**Supplementary Figure 12.** The scatter plots of the association between genetic predicted epilepsy and subtypes on structural connectivity in MR analysis.

**Supplementary Table 1** GWAS summary data of epilepsy and subtypes at P-value<5.0×${10}^{-6}$

To present the more comprehensive data, GWAS summary data of epilepsy and subtypes at P-value < 5.0x can be found in **sheet 1-10** of Supplementary table 1.xlsx

<https://figshare.com/ndownloader/files/50071068?private_link=fc22c4a8a49ae9b9997c>

**Supplementary Table 2** GWAS summary data of functional and structural connectivity at P-value<5.0×${10}^{-6}$

To present the more comprehensive data, GWAS summary data of epilepsy and subtypes at P-value < 5.0x can be found in **sheet 1-14** of Supplementary table 2.xlsx

<https://figshare.com/ndownloader/files/50071059?private_link=fc22c4a8a49ae9b9997c>

**Supplementary Table 3** The F-statistics for the genetic instruments and the power for MR.

| **Exposure** | **Sample size** | **nIV** | **F_stat** |
| --- | --- | --- | --- |
| CAE | 43485 | 12 | 26.11326 |
| GGE | 49388 | 71 | 30.62769 |
| all_epilepsy | 69995 | 44 | 25.69093 |
| focal_epilepsy | 57375 | 28 | 23.32698 |
| GTCS | 42921 | 4 | 24.33189 |
| JAE | 43098 | 9 | 23.05505 |
| JME | 44168 | 36 | 24.56306 |
| focal_HS | 43696 | 13 | 23.27279 |
| focal_lesion_negative | 48214 | 5 | 24.71393 |
| focal_other_lesion | 46649 | 4 | 22.53801 |
| FC_Default | 24336 | 20 | 23.87497 |
| FC_Dorsal.Attention | 24336 | 17 | 23.70495 |
| FC_Frontoparietal | 24336 | 22 | 24.12949 |
| FC_Limbic | 24336 | 10 | 24.90407 |
| FC_Somatomotor | 24336 | 14 | 30.83045 |
| FC_Ventral.Attention | 24336 | 7 | 22.76656 |
| FC_Visual | 24336 | 11 | 22.91905 |
| FC_global | 24336 | 8 | 22.89175 |
| SC_Frontoparietal | 23985 | 16 | 23.17180 |
| SC_Limbic | 23985 | 7 | 23.98101 |
| SC_Somatomotor | 23985 | 17 | 23.85419 |
| SC_Ventral.Attention | 23985 | 12 | 23.27388 |
| SC_Visual | 23985 | 20 | 26.26714 |
| SC_global | 23985 | 56 | 35.48044 |

**Supplementary Table 4.** Results for forward MR estimate

To present the more comprehensive data, results for forward MR estimate effect of epilepsy and subtypes on brain connectivity can be found in **sheet 1** of Supplementary table 4-5.xlsx

<https://figshare.com/ndownloader/files/52595834?private_link=fc22c4a8a49ae9b9997c>

**Supplementary Table 5.** Results for reverse MR estimate

To present the more comprehensive data, results for reverse MR estimate effect of brain connectivity on epilepsy and subtypes can be found in **sheet 2** of Supplementary table 4-5.xlsx

<https://figshare.com/ndownloader/files/52595834?private_link=fc22c4a8a49ae9b9997c>

**Supplementary Table 6.** Sensitivity analysis of instrumental variables used in MR analyses

To present the more comprehensive data, sensitivity analysis of instrumental variables used in MR analyses can be found in Supplementary table 6.xlsx

<https://figshare.com/ndownloader/files/52595831?private_link=fc22c4a8a49ae9b9997c>

**Supplementary Table 7.** The result of MR-PRESSO test

To present the more comprehensive data, the result of MR-PRESSO test can be found in Supplementary table 7.xlsx

<https://figshare.com/ndownloader/files/52595840?private_link=fc22c4a8a49ae9b9997c>

**Supplementary Table 8.** The result of MR Steiger test

To present the more comprehensive data, the result of MR Steiger test can be found in Supplementary table 8.xlsx

<https://figshare.com/ndownloader/files/52595837?private_link=fc22c4a8a49ae9b9997c>

**Supplementary Table 9.** Annotated SNP list by LDTrait tool

To present the more comprehensive data, annotated SNP list by LDTrait tool can be found in **sheet 1-6** of Supplementary table 9.xlsx

<https://figshare.com/ndownloader/files/52595843?private_link=fc22c4a8a49ae9b9997c>

**Supplementary Table 10.** Results of the MVMR analysis for two sample MR screening

To present the more comprehensive data, results of the MVMR analysis for two sample MR screening can be found in Supplementary table 10.xlsx

<https://figshare.com/ndownloader/files/52595846?private_link=fc22c4a8a49ae9b9997c>

**Supplementary Table 11.** Results of the FUMA analysis for functional annotation

To present the more comprehensive data, results of the FUMA analysis for functional annotation can be found in Supplementary table 11.zip

<https://figshare.com/ndownloader/files/52627169?private_link=fc22c4a8a49ae9b9997c>

**Supplementary Figure 1** The forest plots of the association between genetic predicted epilepsy and subtypes on functional connectivity in MR analysis.

Supplementary Figure 1 (a). Single SNP analysis for individual and combined SNP effects of GGE on somatomotor.


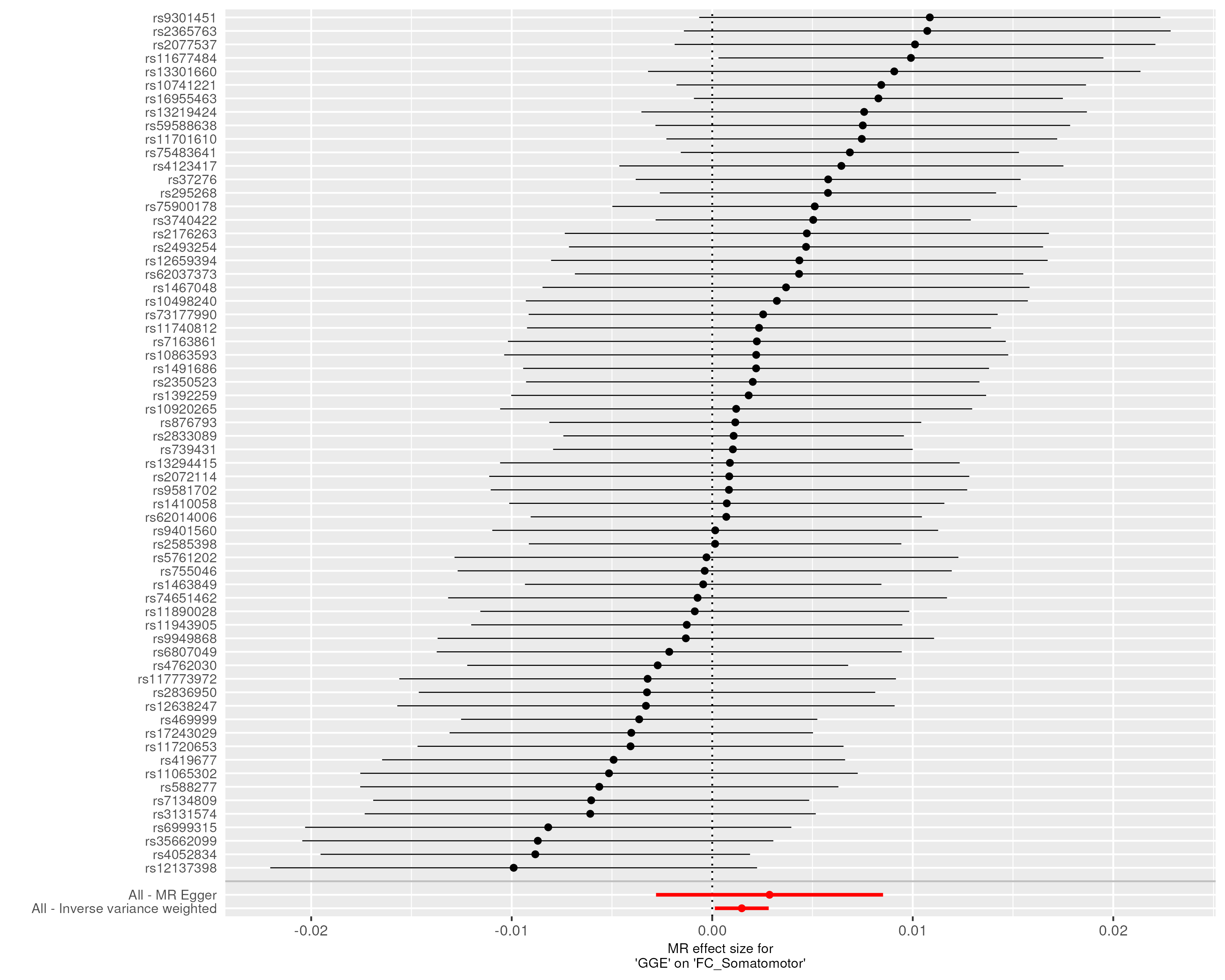


Supplementary Figure 1 (b). Single SNP analysis for individual and combined SNP effects of GGE on limbic.


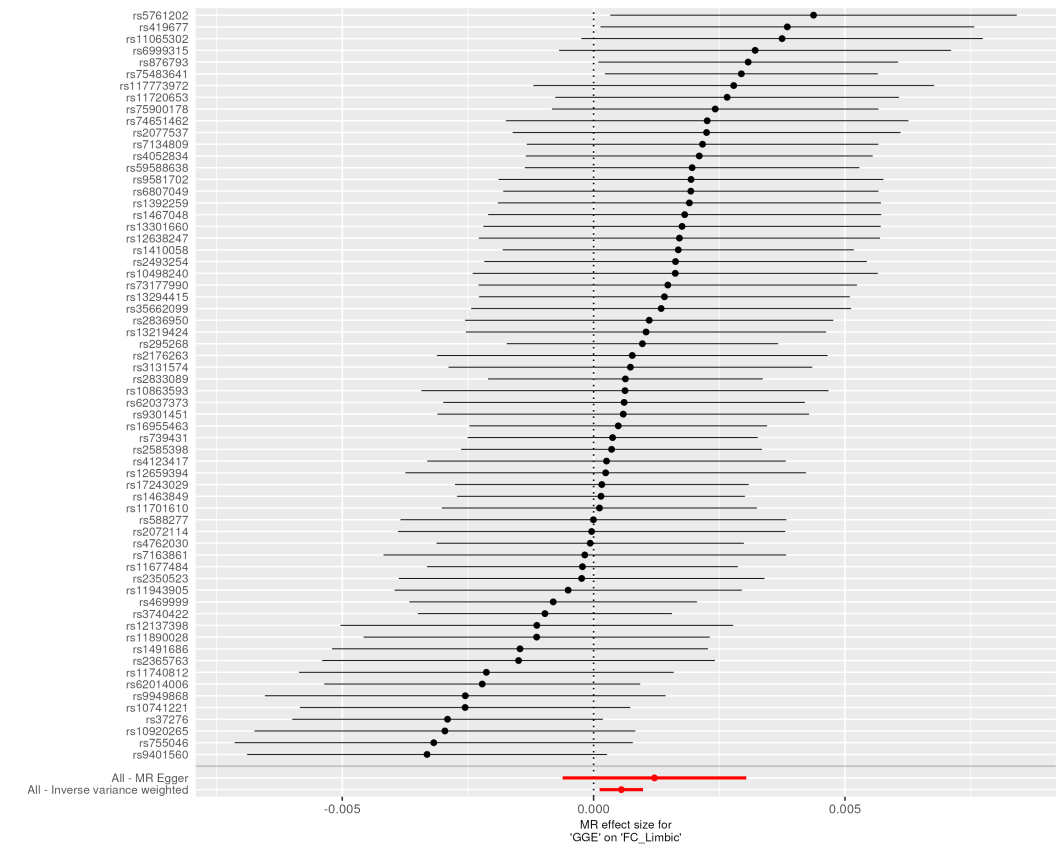


Supplementary Figure 1 (c). Single SNP analysis for individual and combined SNP effects of GGE on dorsal attention.


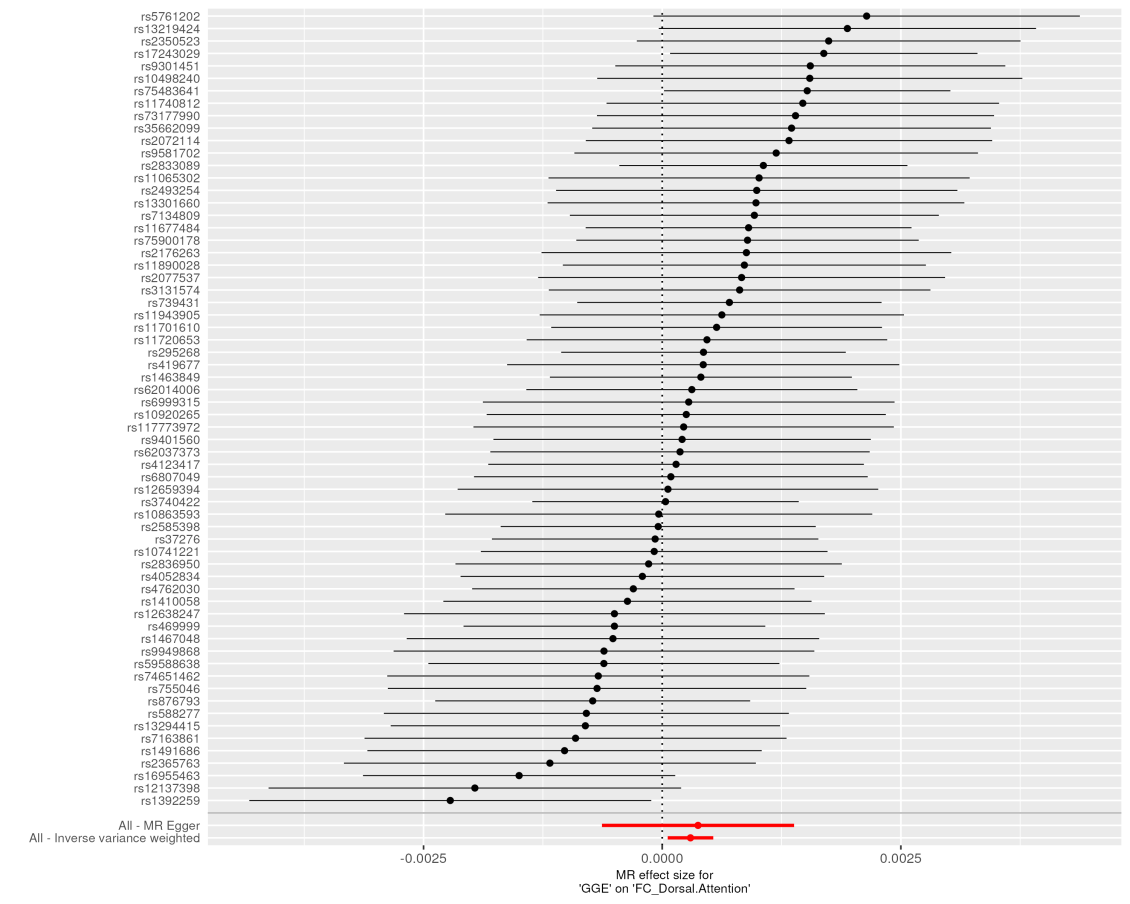


Supplementary Figure 1 (d). Single SNP analysis for individual and combined SNP effects of GGE on default.


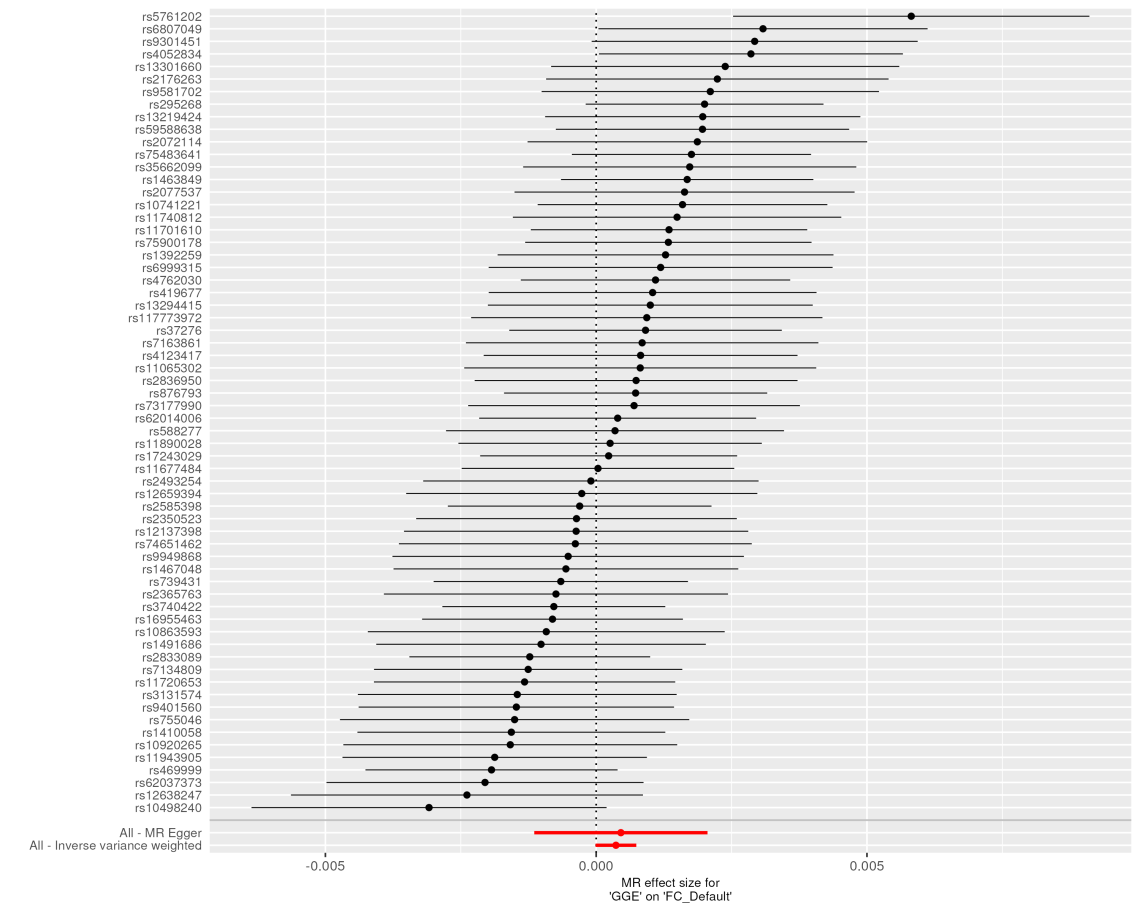


Supplementary Figure 1 (e). Single SNP analysis for individual and combined SNP effects of focal_lesion_negative on frontoparietal.


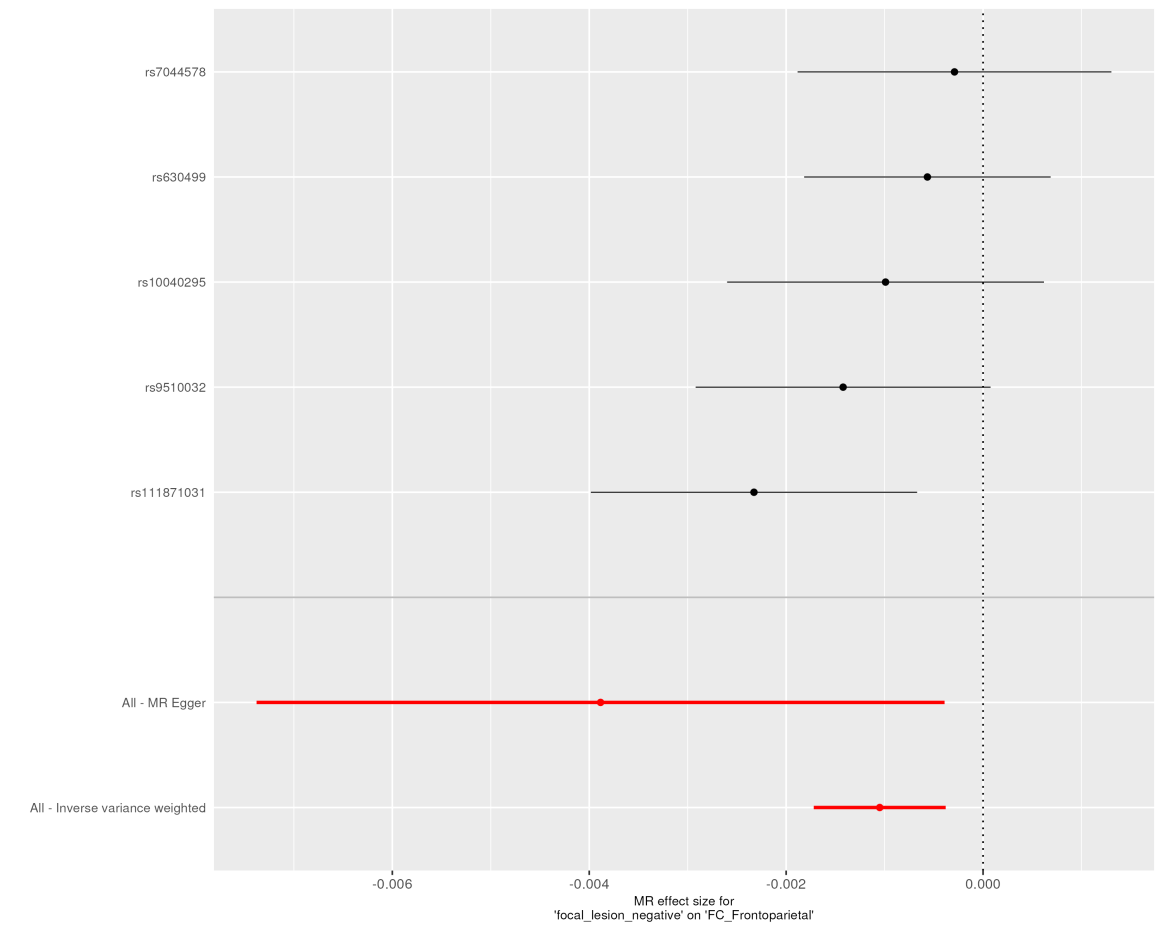


Supplementary Figure 1 (f). Single SNP analysis for individual and combined SNP effects of focal epilepsy on dorsal attention.


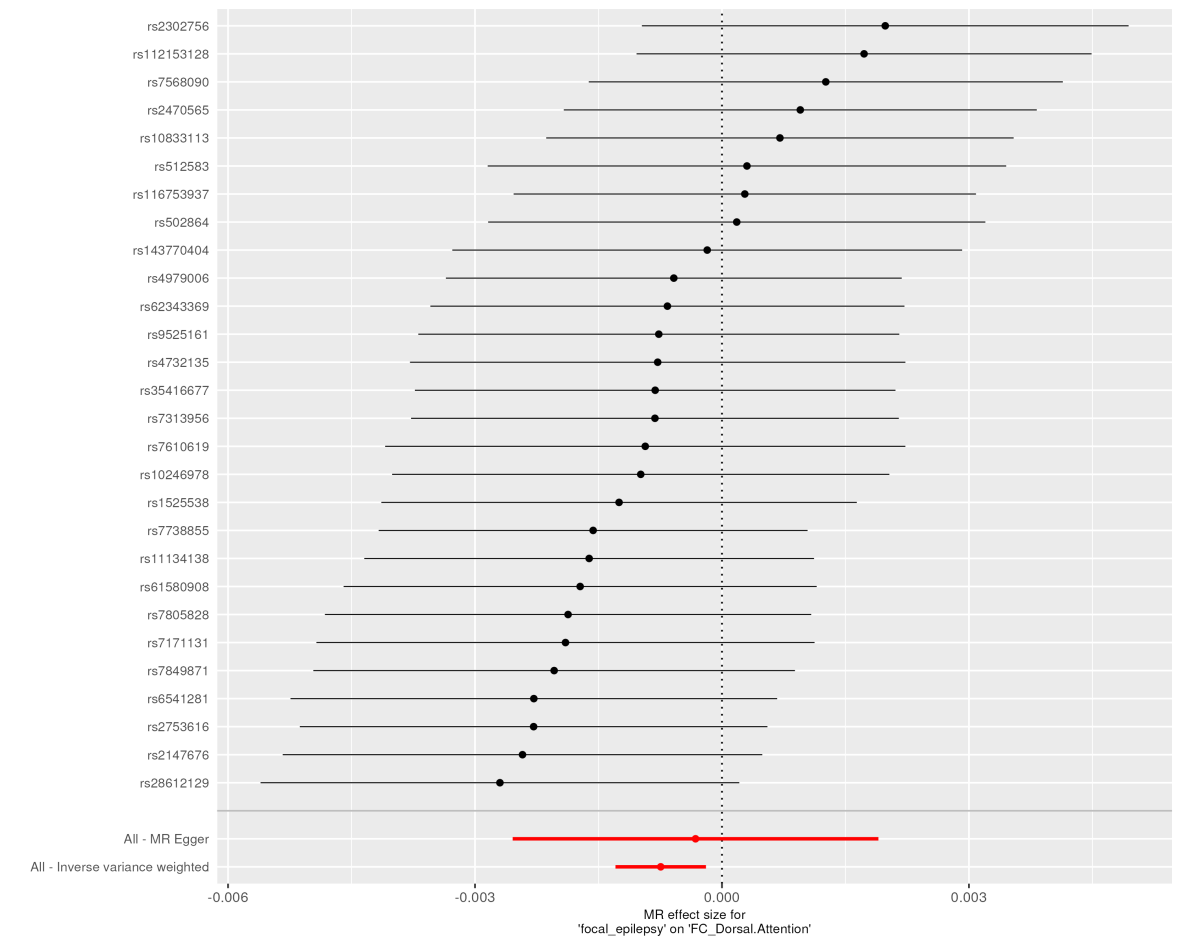


**Supplementary Figure 2** The funnel plots of the association between genetic predicted epilepsy and subtypes on functional connectivity in MR analysis.

Supplementary Figure 2 (a). The funnel plots of the association between genetic predicted focal epilepsy on dorsal attention in MR analysis.


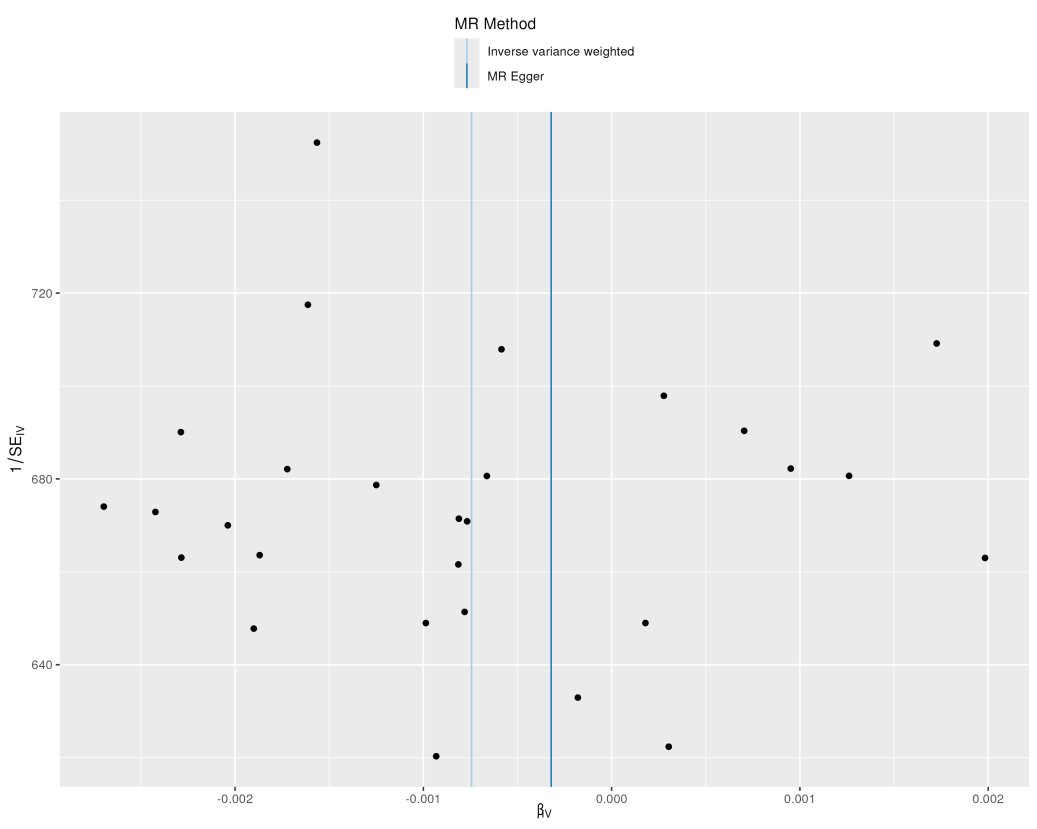


Supplementary Figure 2 (b). The funnel plots of the association between genetic predicted focal lesion negative on frontoparietal in MR analysis.


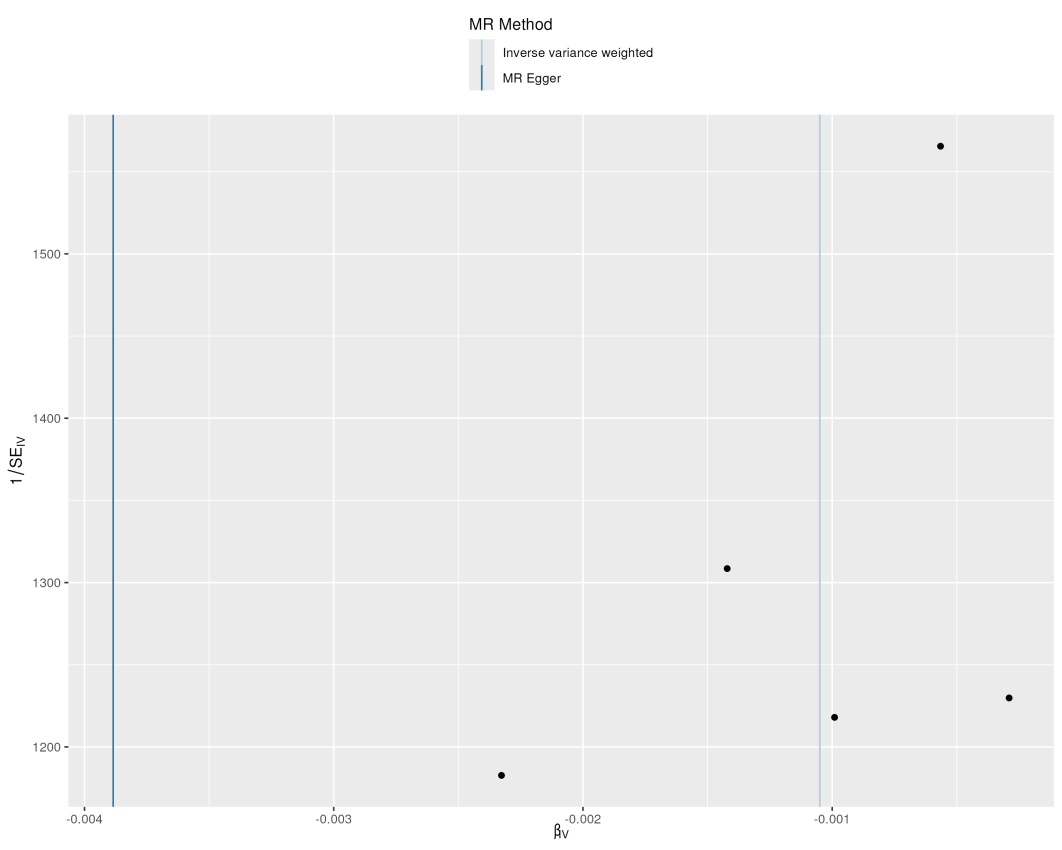


Supplementary Figure 2 (c). The funnel plots of the association between genetic predicted GGE on default in MR analysis.

**
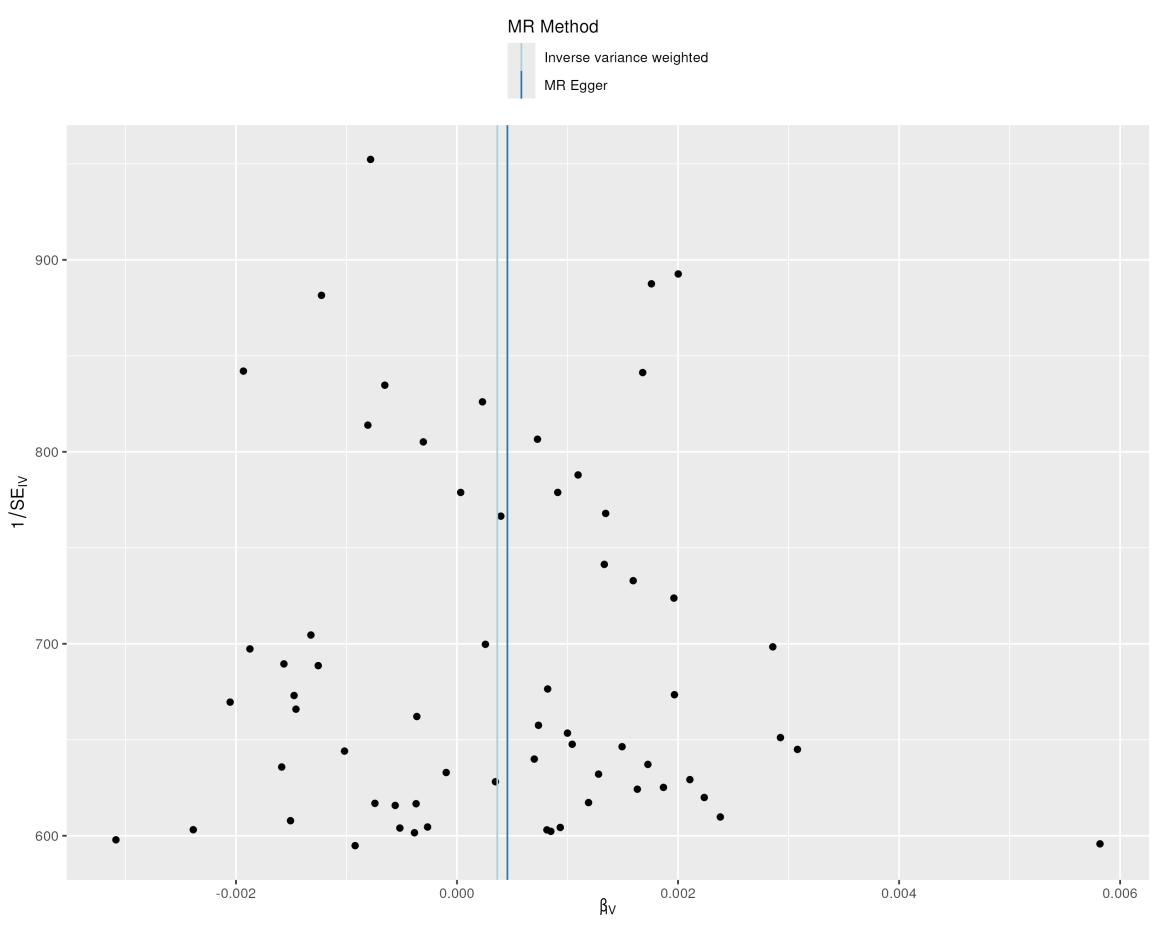
**

Supplementary Figure 2 (d). The funnel plots of the association between genetic predicted GGE on dorsal attention in MR analysis.

**
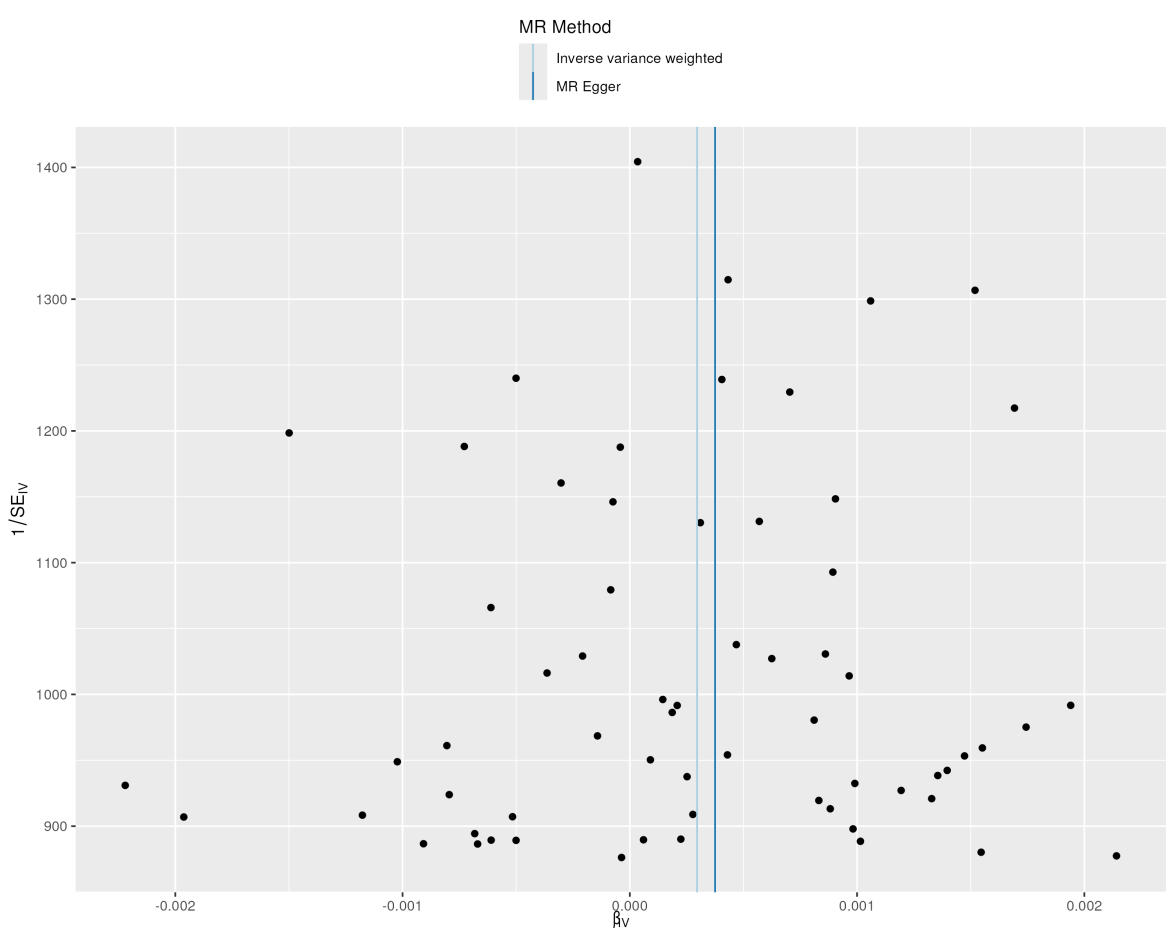
**

Supplementary Figure 2 (e). The funnel plots of the association between genetic predicted GGE on limbic in MR analysis.

**
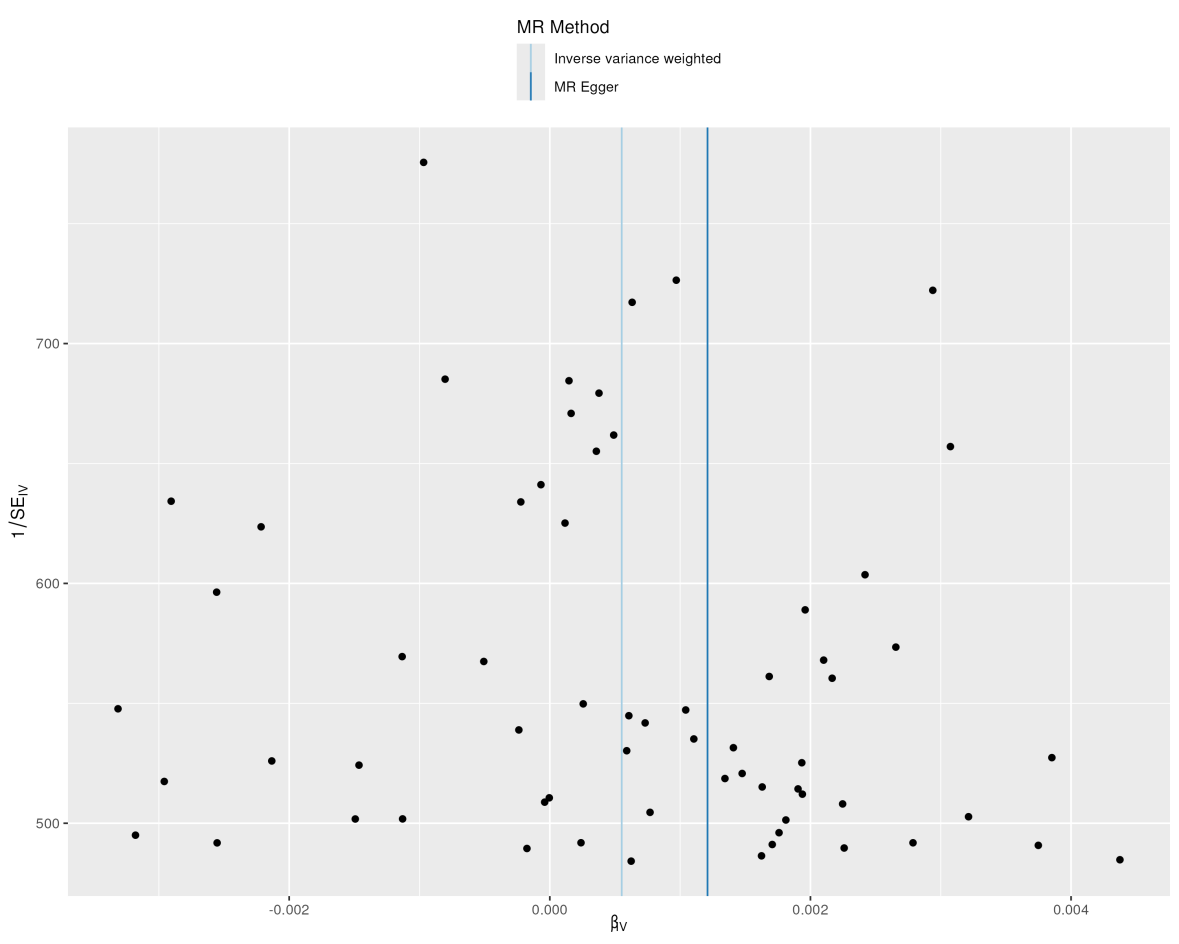
**

Supplementary Figure 2 (f). The funnel plots of the association between genetic predicted GGE on somatomotor in MR analysis.

**
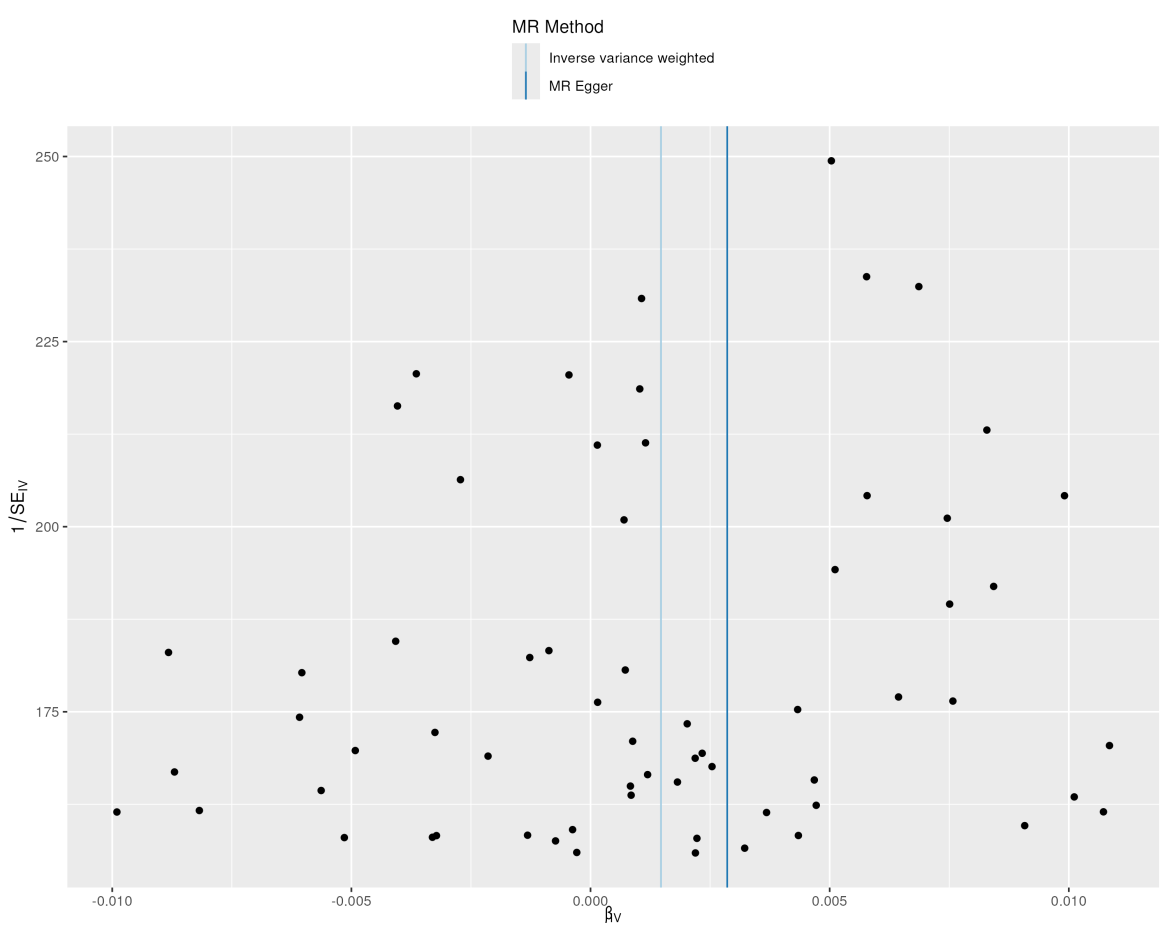
**

**Supplementary Figure 3** The leave-one-out analysis of the association between genetic predicted epilepsy and subtypes on functional connectivity in MR analysis.

Supplementary Figure 3 (a). The leave-one-out analysis of the association between genetic predicted focal epilepsy on dorsal attention in MR analysis.

**
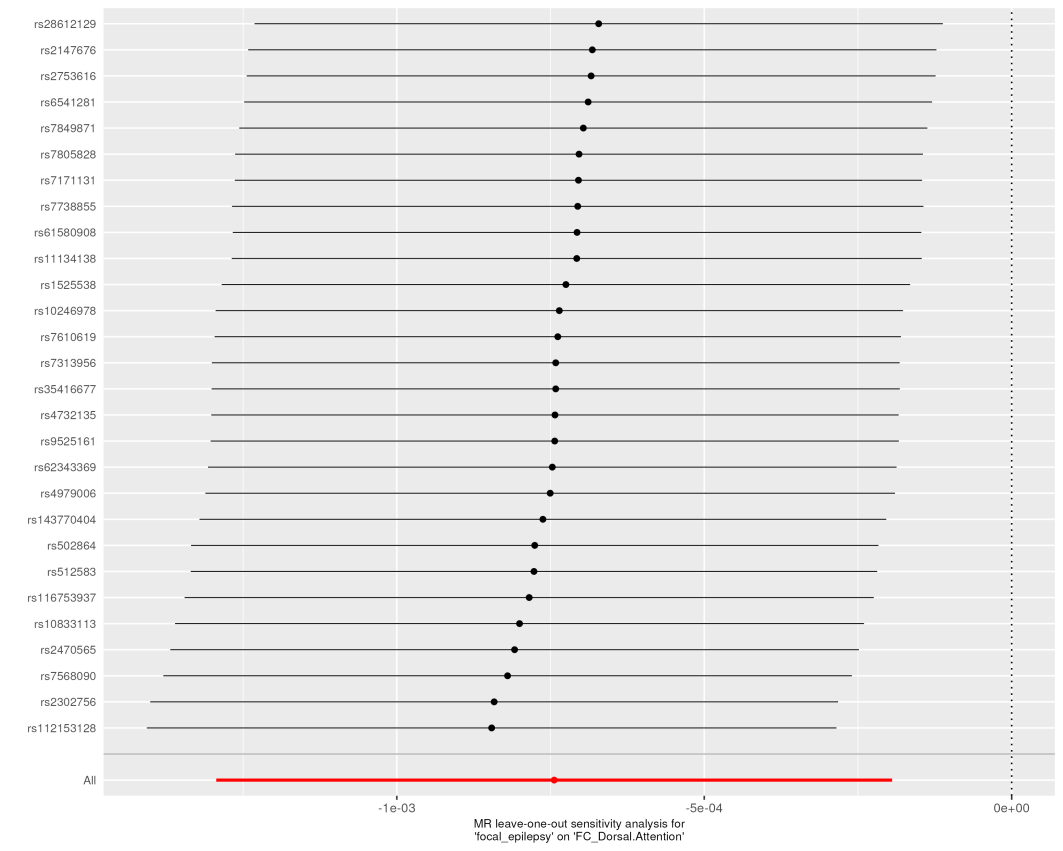
**

Supplementary Figure 3 (b). The leave-one-out analysis of the association between genetic predicted GGE on default in MR analysis.

**
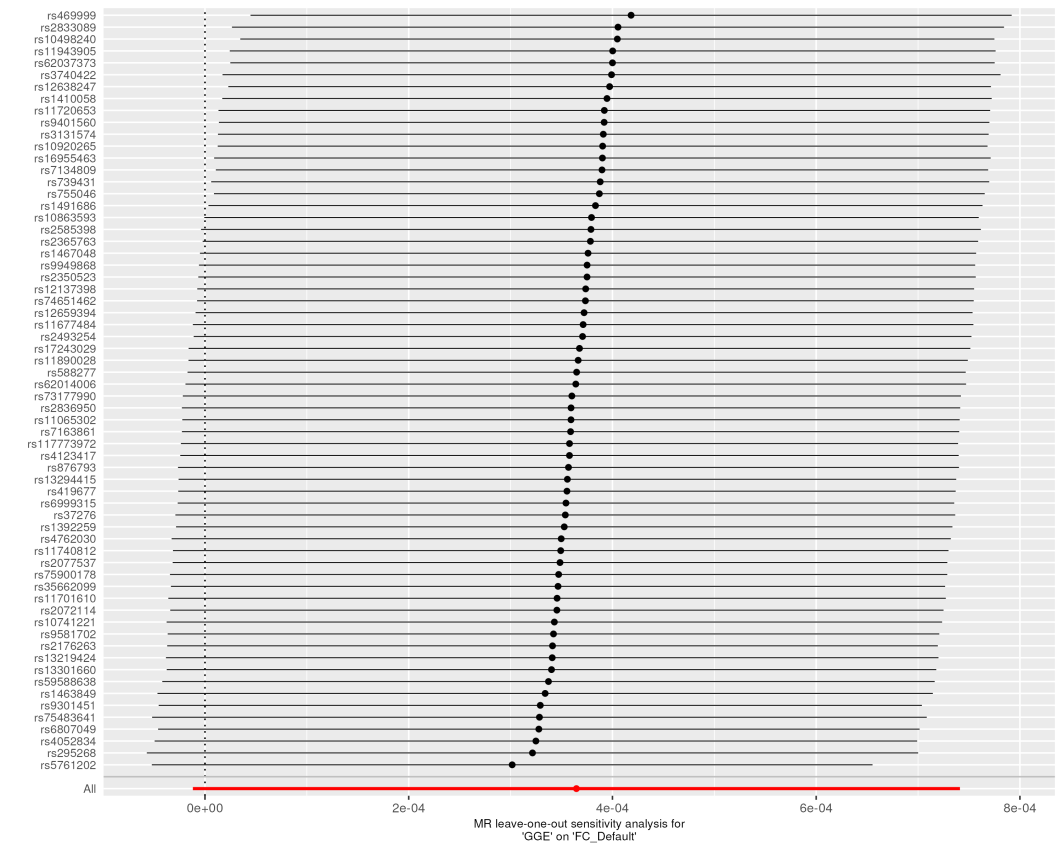
**

Supplementary Figure 3 (c). The leave-one-out analysis of the association between genetic predicted GGE on dorsal attention in MR analysis.

**
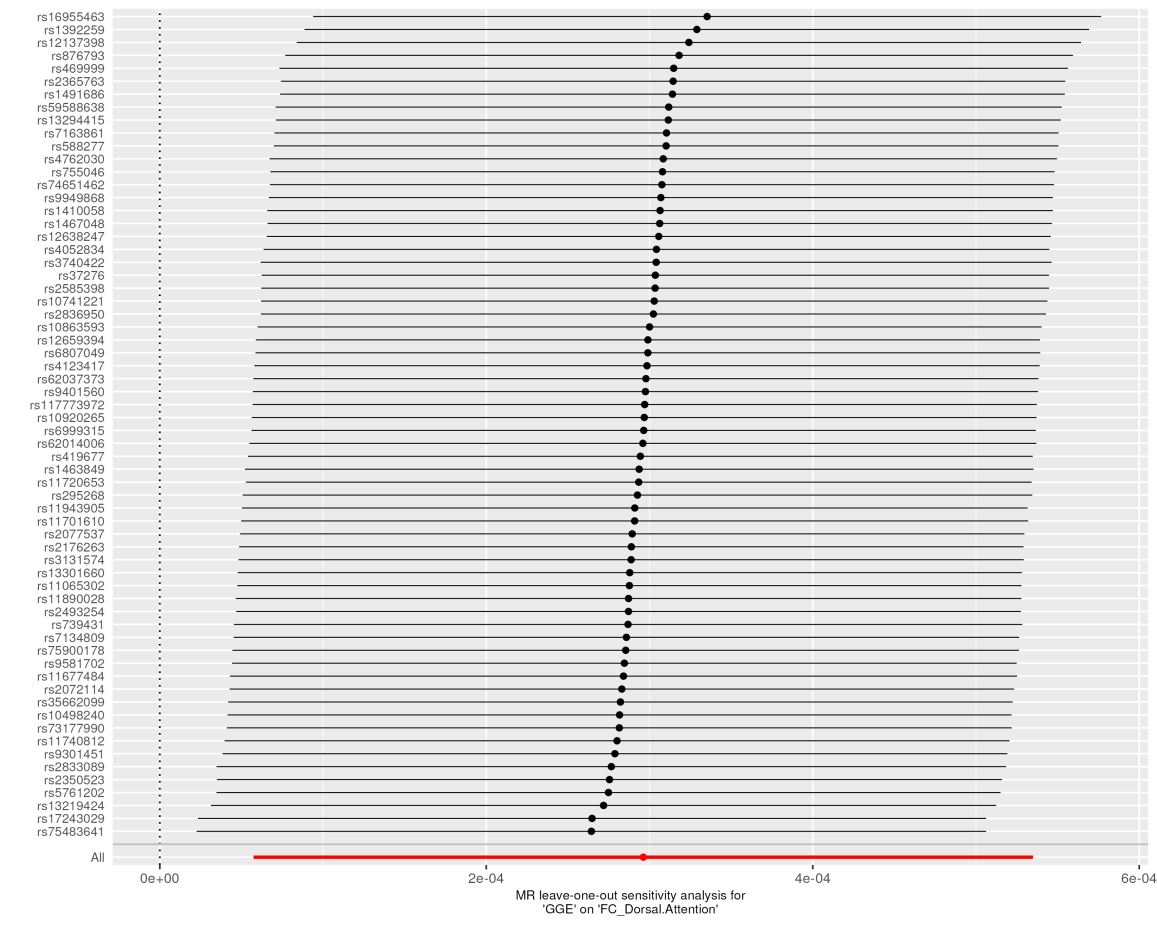
**

Supplementary Figure 3 (d). The leave-one-out analysis of the association between genetic predicted GGE on limbic in MR analysis.

**
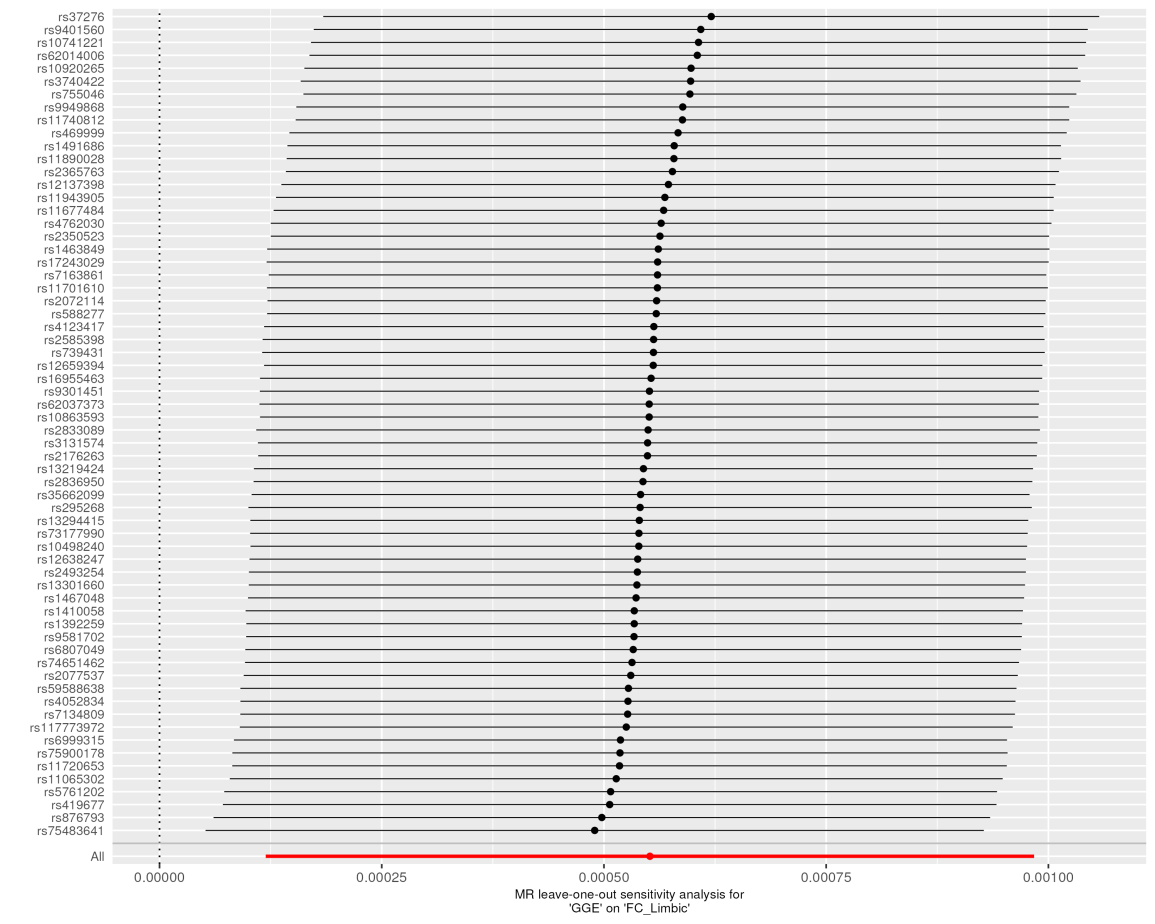
**

Supplementary Figure 3 (e). The leave-one-out analysis of the association between genetic predicted GGE on somatomotor in MR analysis.

**
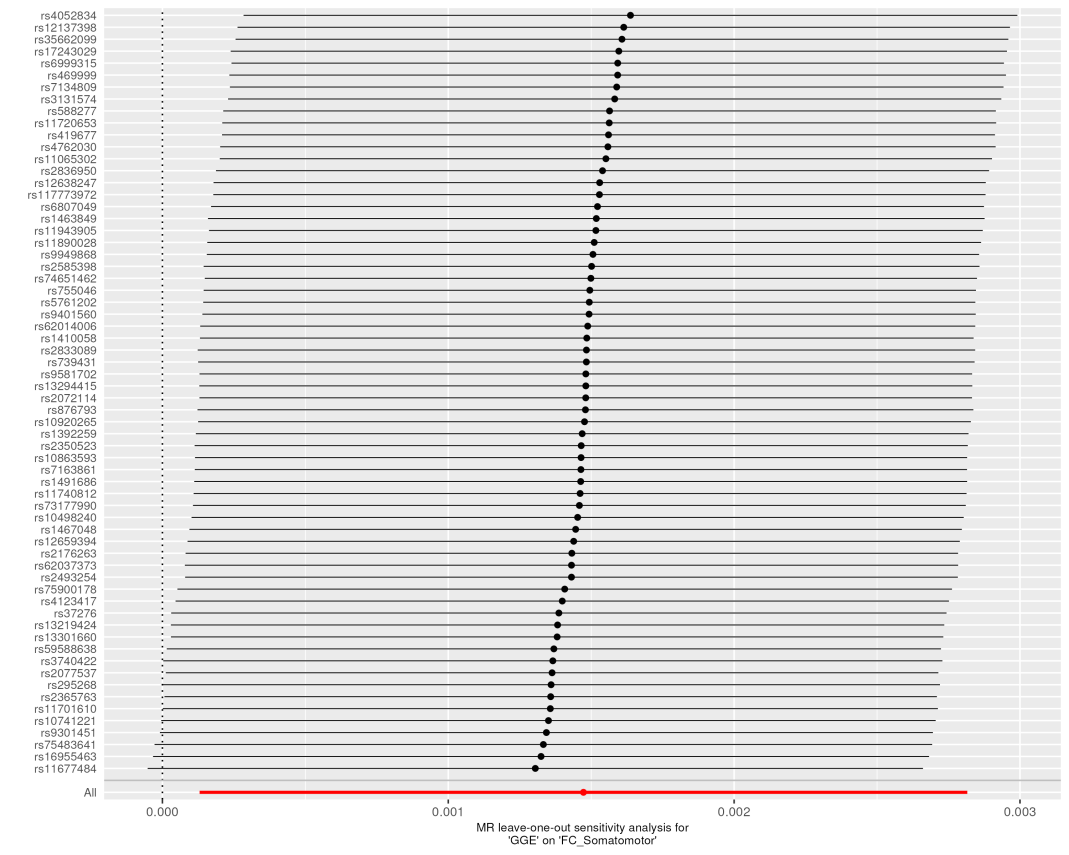
**

**Supplementary Figure 4** The scatter plots of the association between genetic predicted epilepsy and subtypes on functional connectivity in MR analysis.

Supplementary Figure 4 (a). The scatter plots of the association between genetic predicted focal epilepsy on dorsal attention in MR analysis.


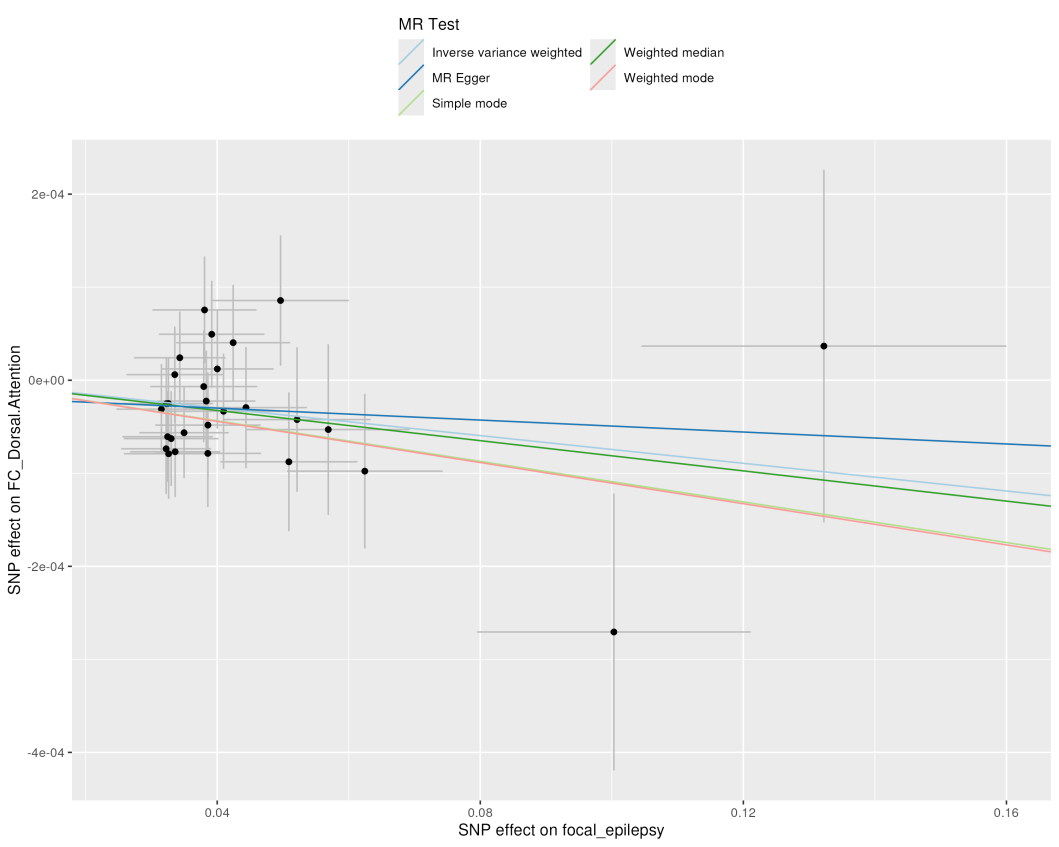


Supplementary Figure 4 (b). The scatter plots of the association between genetic predicted focal lesion negative on frontoparietal in MR analysis.


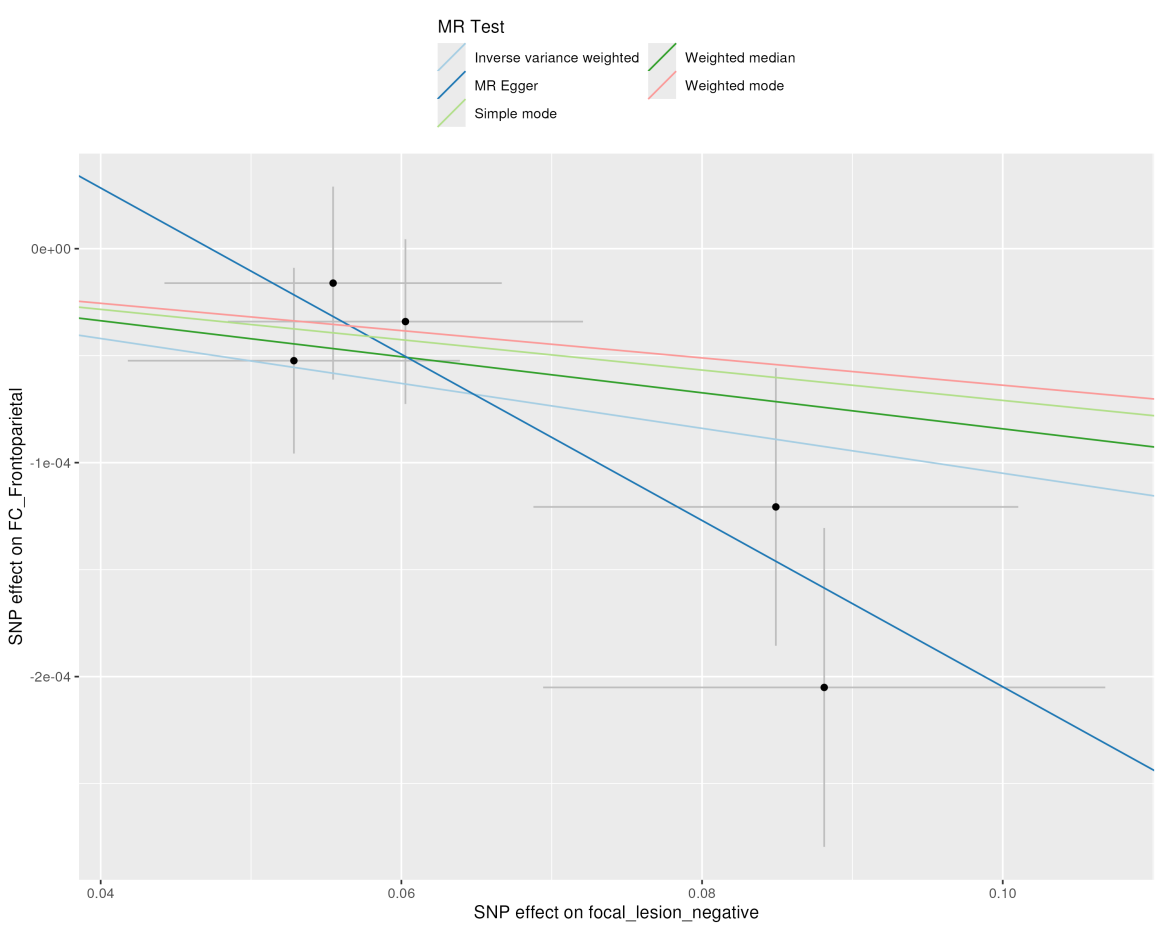


Supplementary Figure 4 (c). The scatter plots of the association between genetic predicted GGE on default in MR analysis.


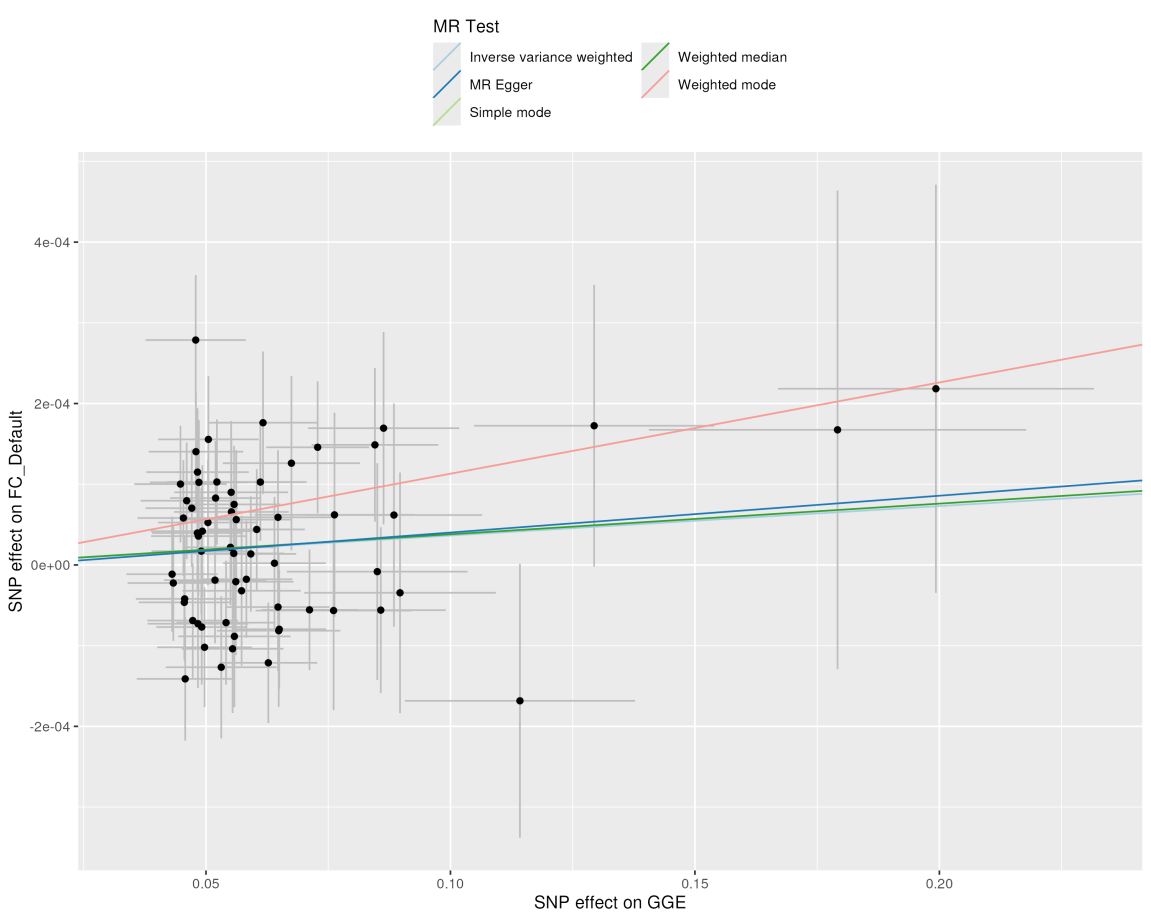


Supplementary Figure 4 (d). The scatter plots of the association between genetic predicted GGE on dorsal attention in MR analysis.


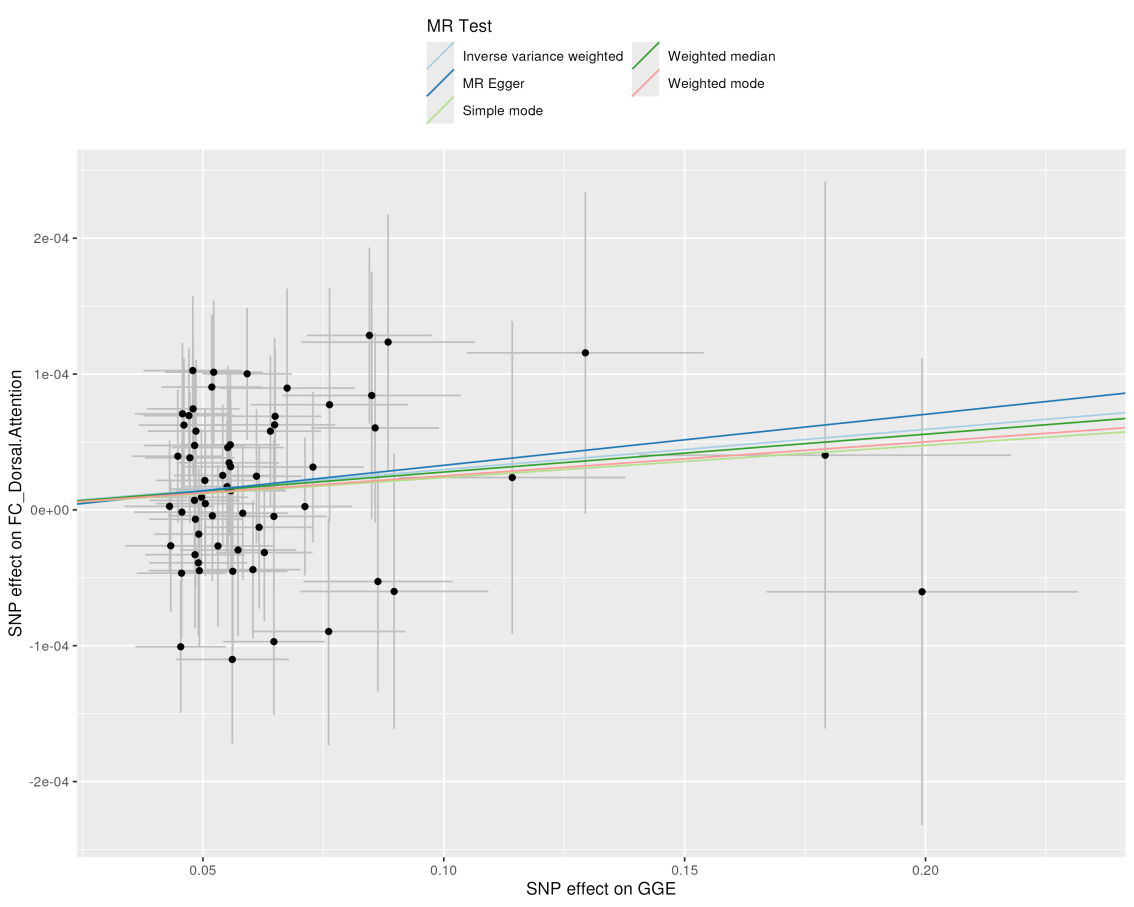


Supplementary Figure 4 (e). The scatter plots of the association between genetic predicted GGE on limbic in MR analysis.


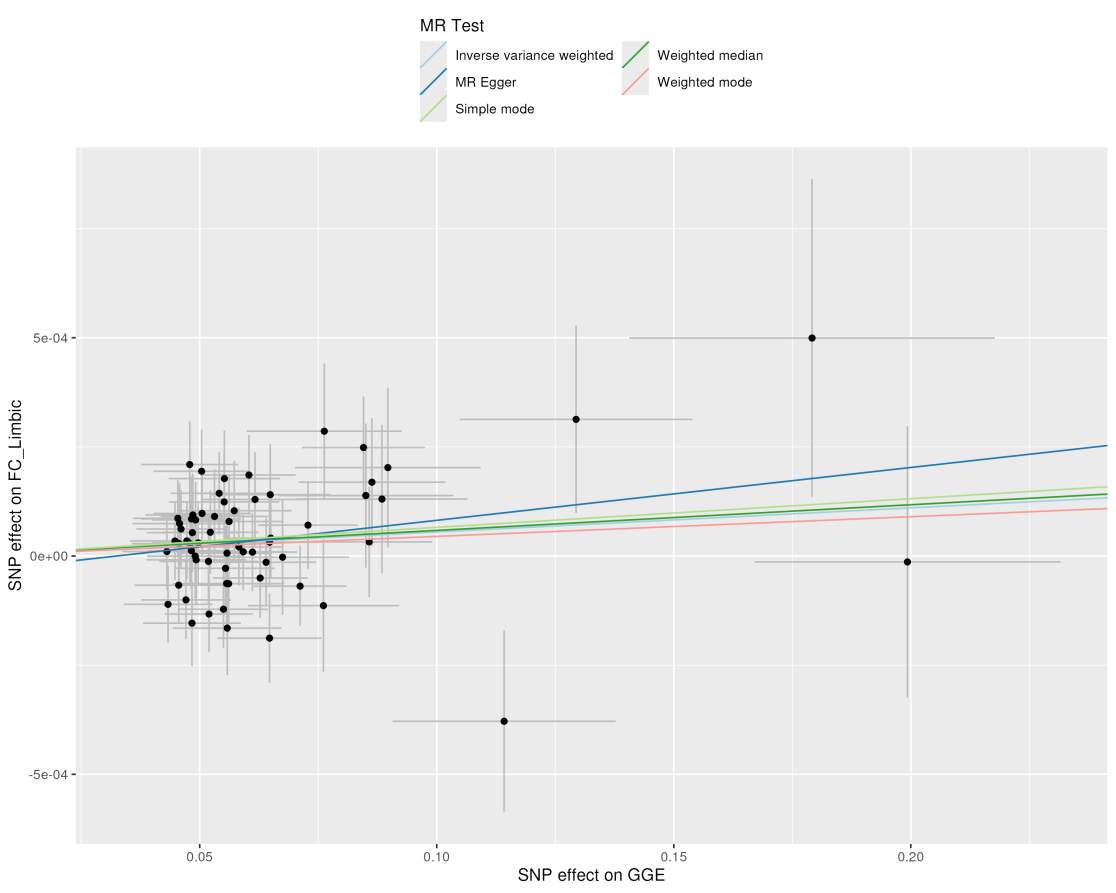


Supplementary Figure 4 (f). The scatter plots of the association between genetic predicted GGE on somatomotor in MR analysis.


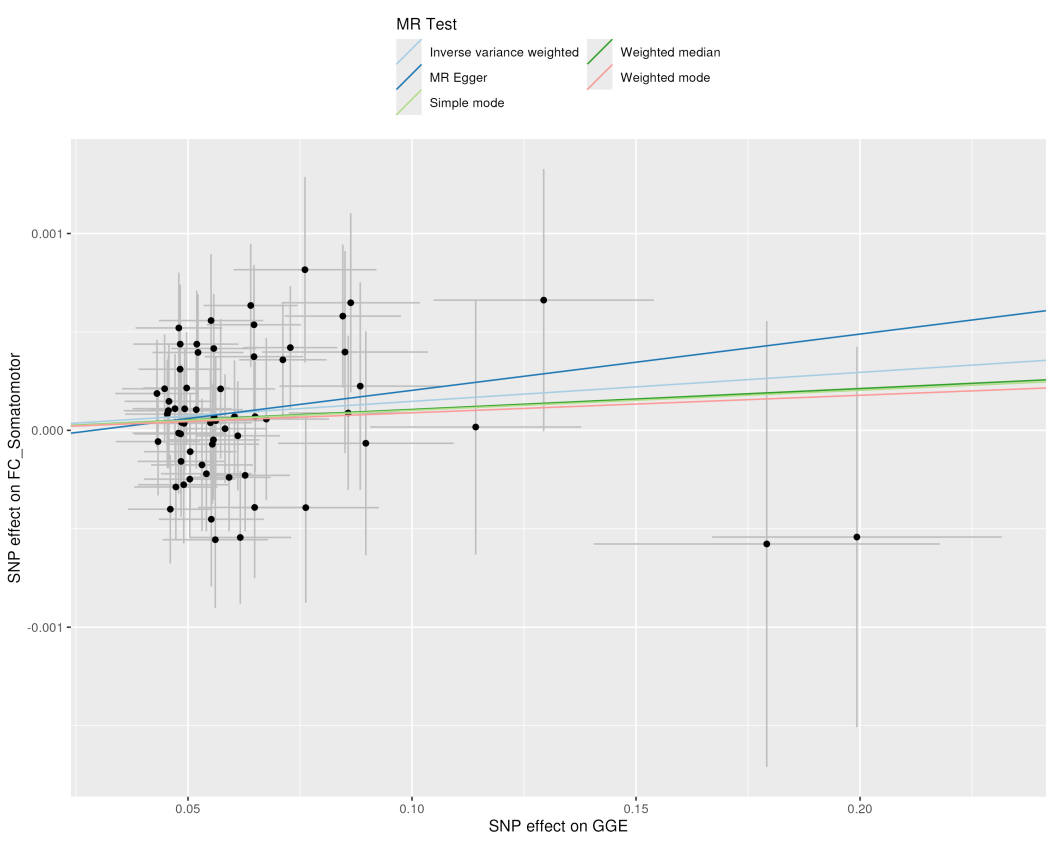


**Supplementary Figure 5** The forest plots of the association between genetic predicted epilepsy and subtypes on structural connectivity in MR analysis.

Supplementary Figure 5 (a). Single SNP analysis for individual and combined SNP effects of focal epilepsy on global.


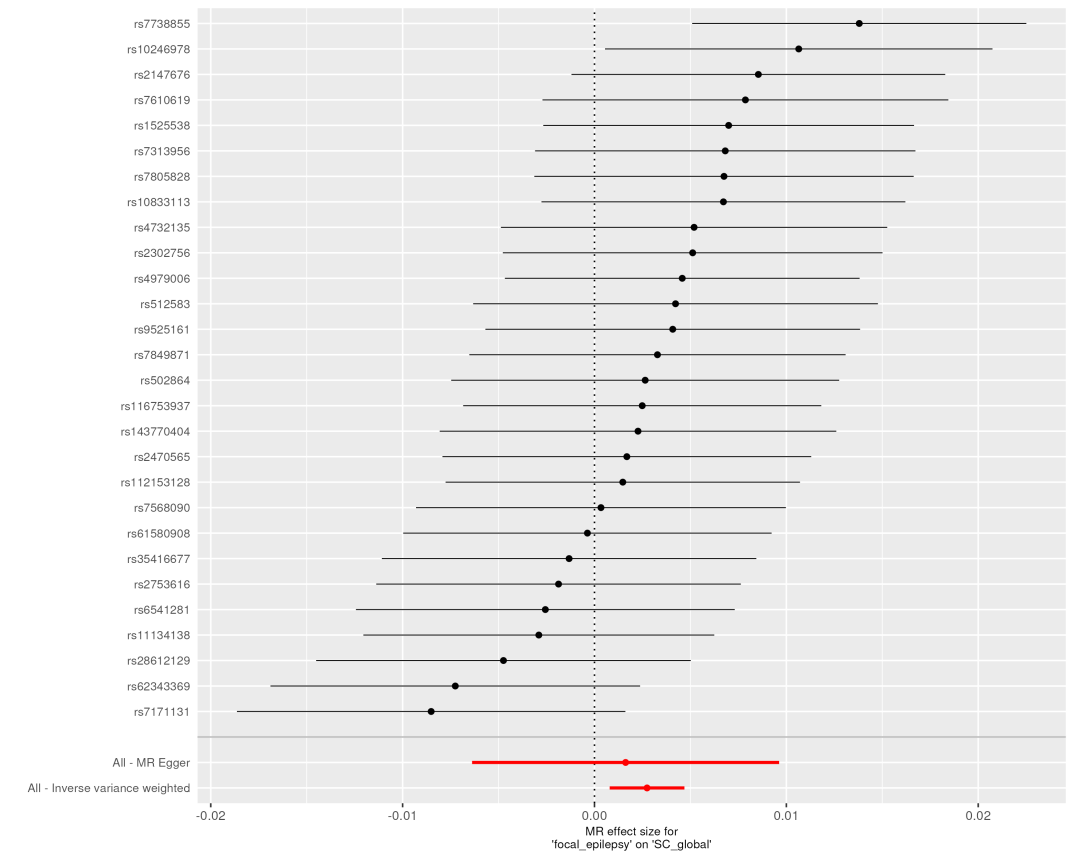


Supplementary Figure 5 (b). Single SNP analysis for individual and combined SNP effects of CAE on ventral attention.


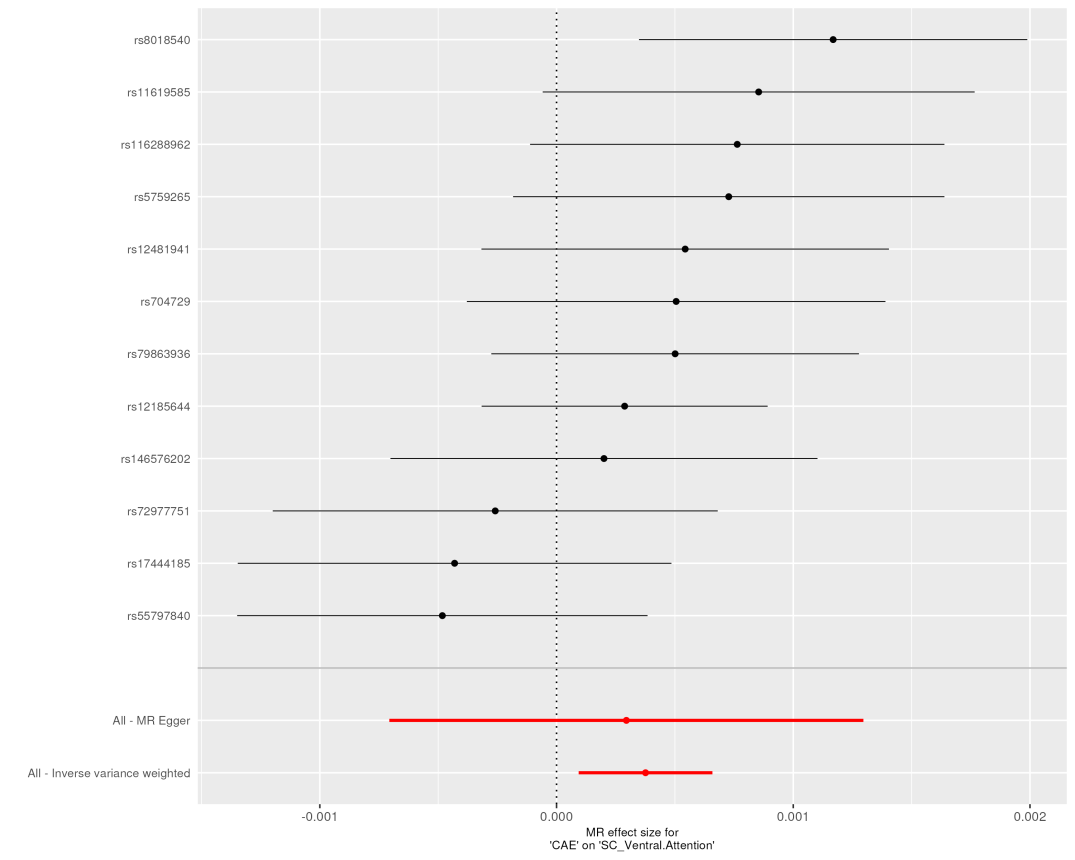


**Supplementary Figure 6** The funnel plots of the association between genetic predicted epilepsy and subtypes on structural connectivity in MR analysis.

Supplementary Figure 6 (a). The funnel plots of the association between genetic predicted CAE on ventral attention in MR analysis.


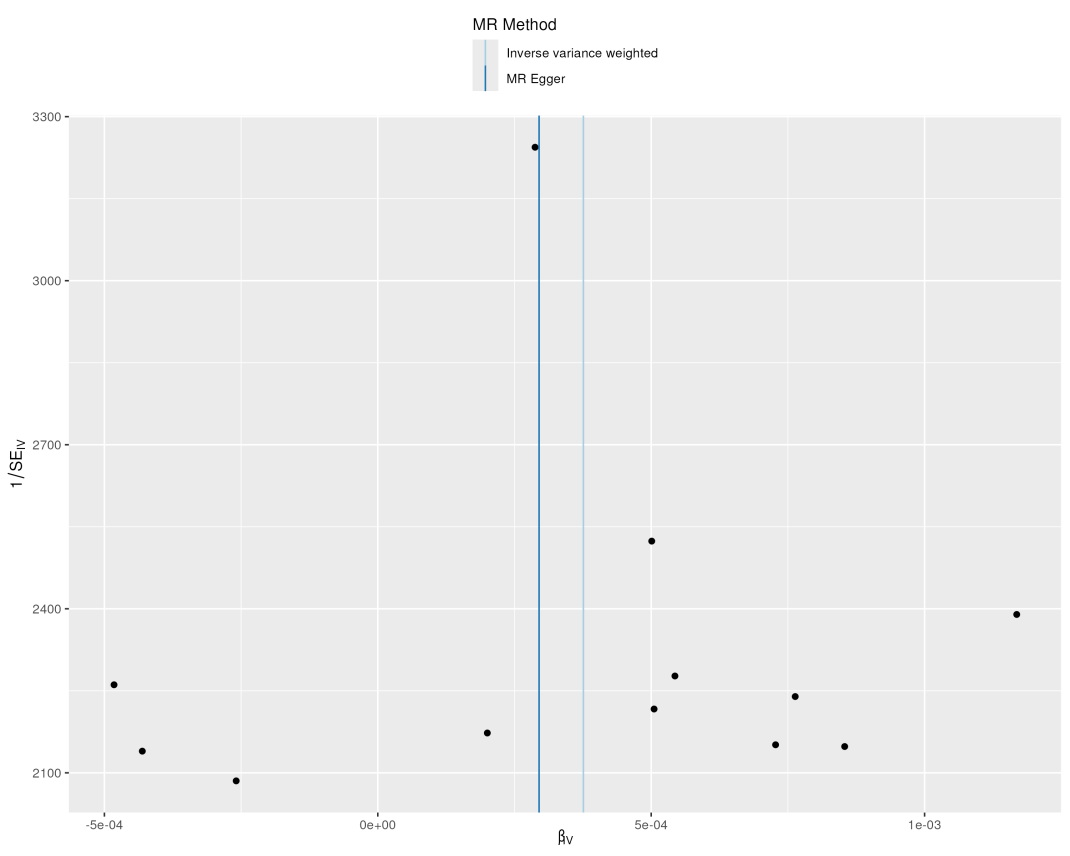


Supplementary Figure 6 (b). The funnel plots of the association between genetic predicted focal epilepsy on global in MR analysis.


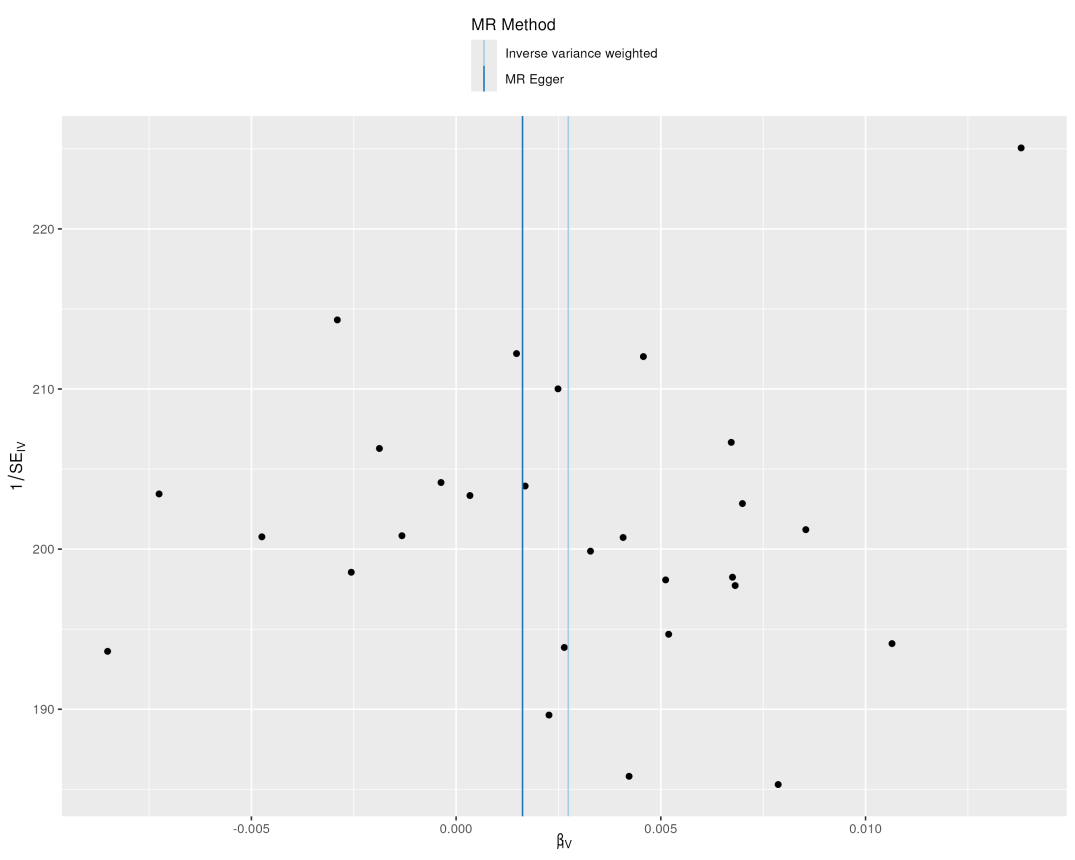


**Supplementary Figure 7** The leave-one-out analysis of the association between genetic predicted epilepsy and subtypes on structural connectivity in MR analysis.

Supplementary Figure 7 (a). The leave-one-out analysis of the association between genetic predicted CAE on ventral attention in MR analysis.


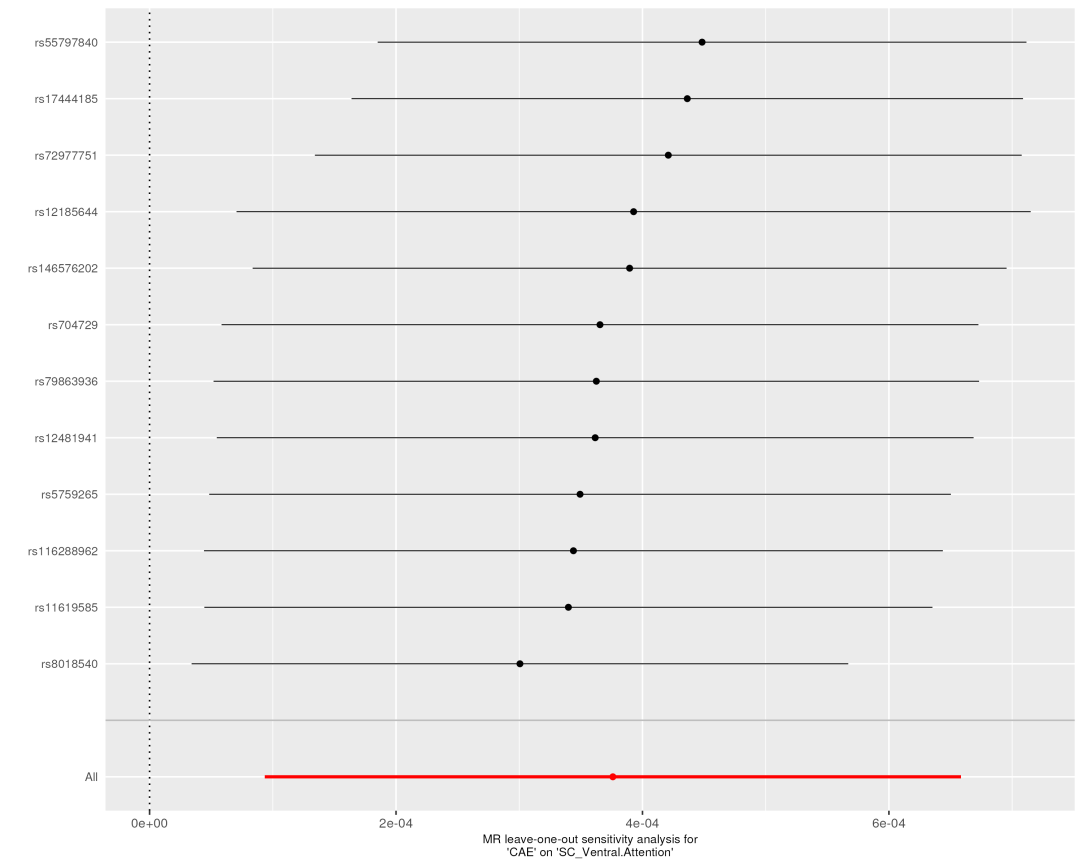


Supplementary Figure 7 (b). The leave-one-out analysis of the association between genetic predicted focal epilepsy on global in MR analysis.


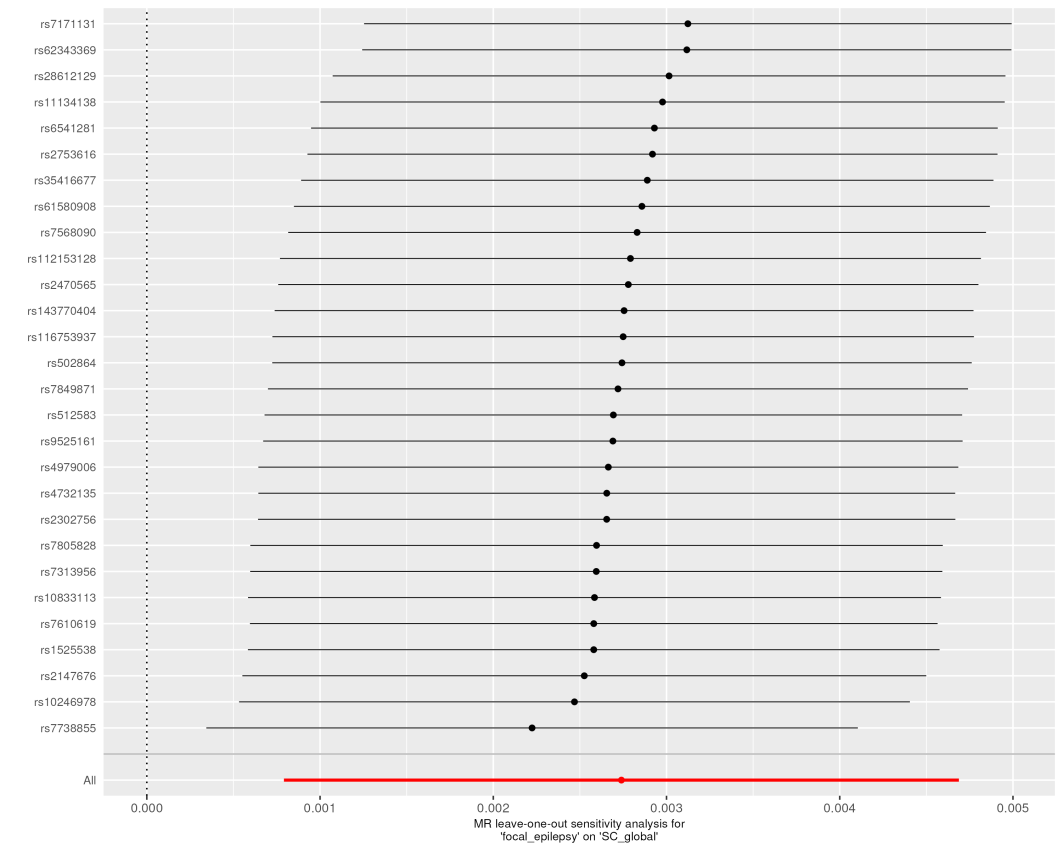


**Supplementary Figure 8** The scatter plots of the association between genetic predicted epilepsy and subtypes on structural connectivity in MR analysis.

Supplementary Figure 8 (a). The scatter plots of the association between genetic predicted CAE on ventral attention in MR analysis.


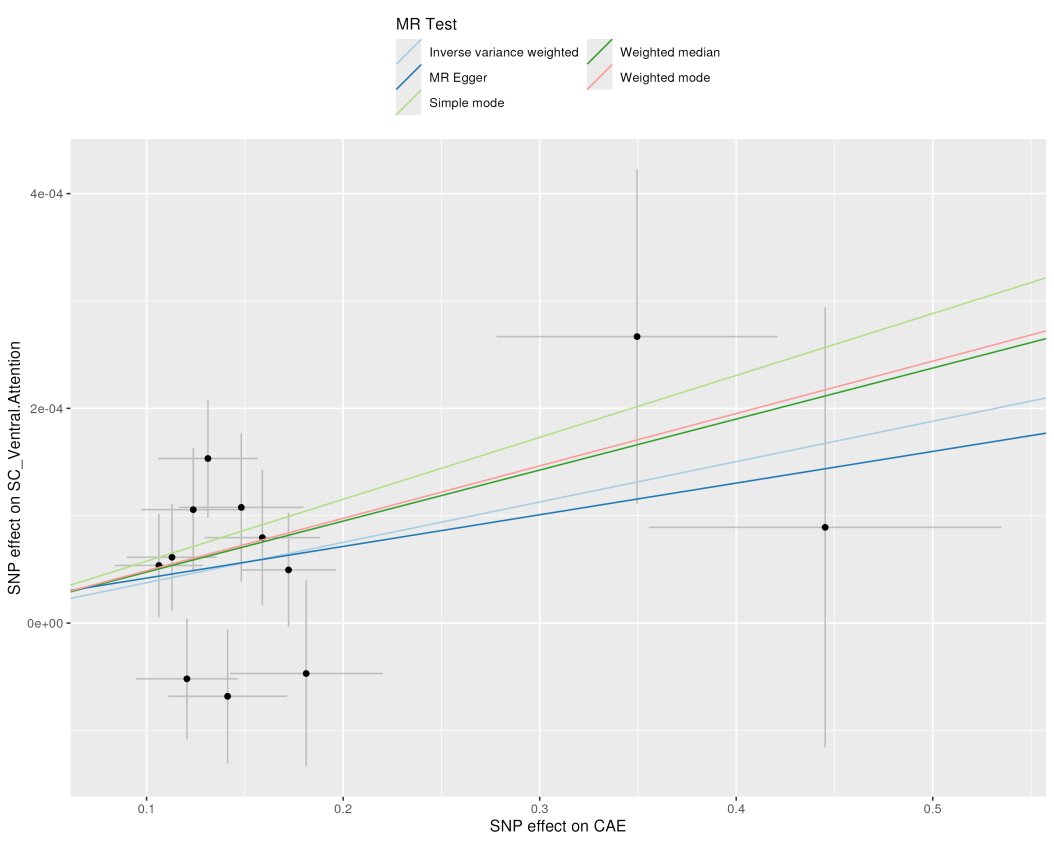


Supplementary Figure 8 (b). The scatter plots of the association between genetic predicted focal epilepsy on global in MR analysis.


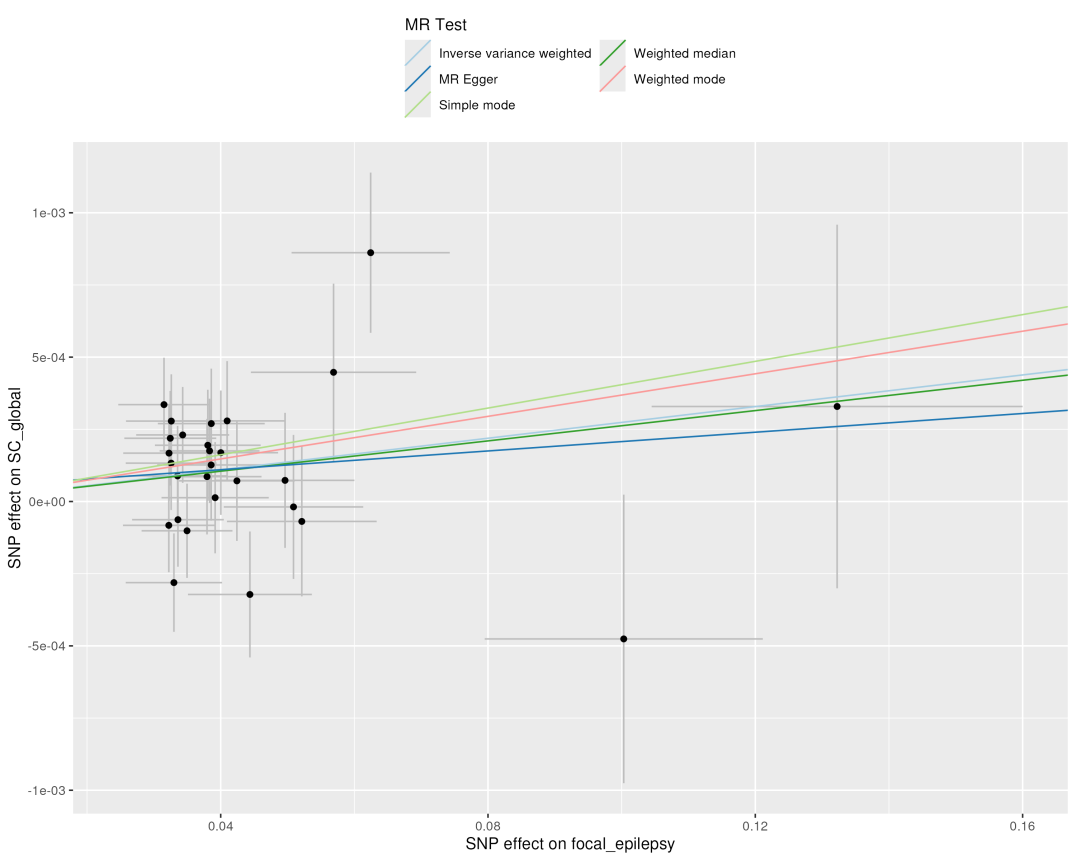


**Supplementary Figure 9** The forest plots of the association between genetic predicted structural connectivity on epilepsy and subtypes in MR analysis.

Supplementary Figure 9 (a). Single SNP analysis for individual and combined SNP effects of frontoparietal on CAE.


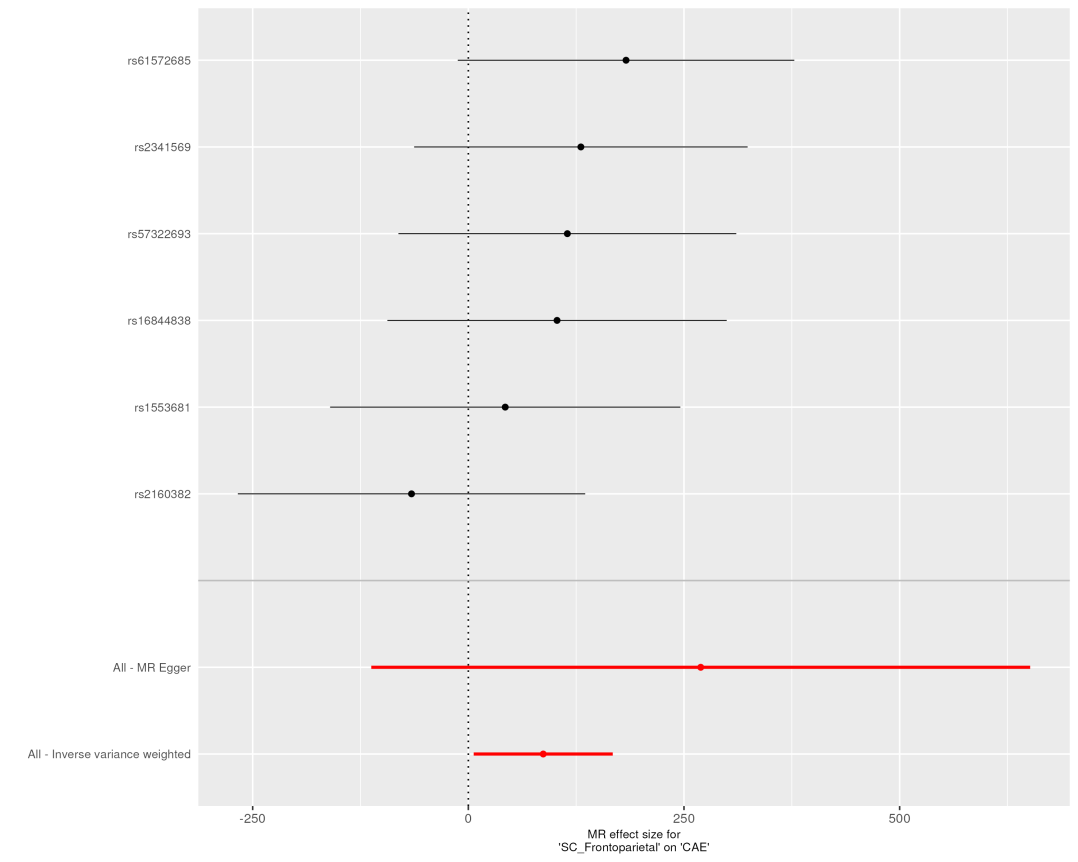


Supplementary Figure 9 (b). Single SNP analysis for individual and combined SNP effects of global on all epilepsy.


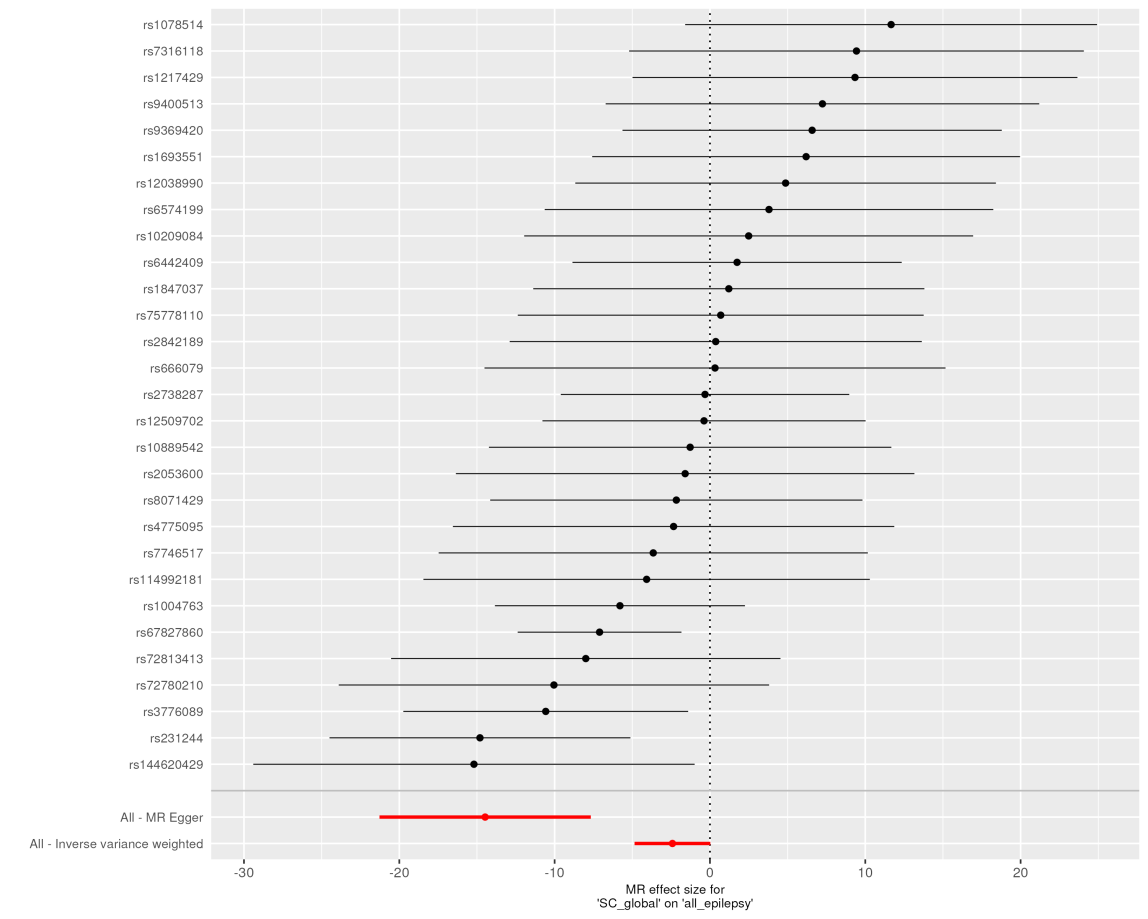


Supplementary Figure 9 (c). Single SNP analysis for individual and combined SNP effects of visual on GTCS.


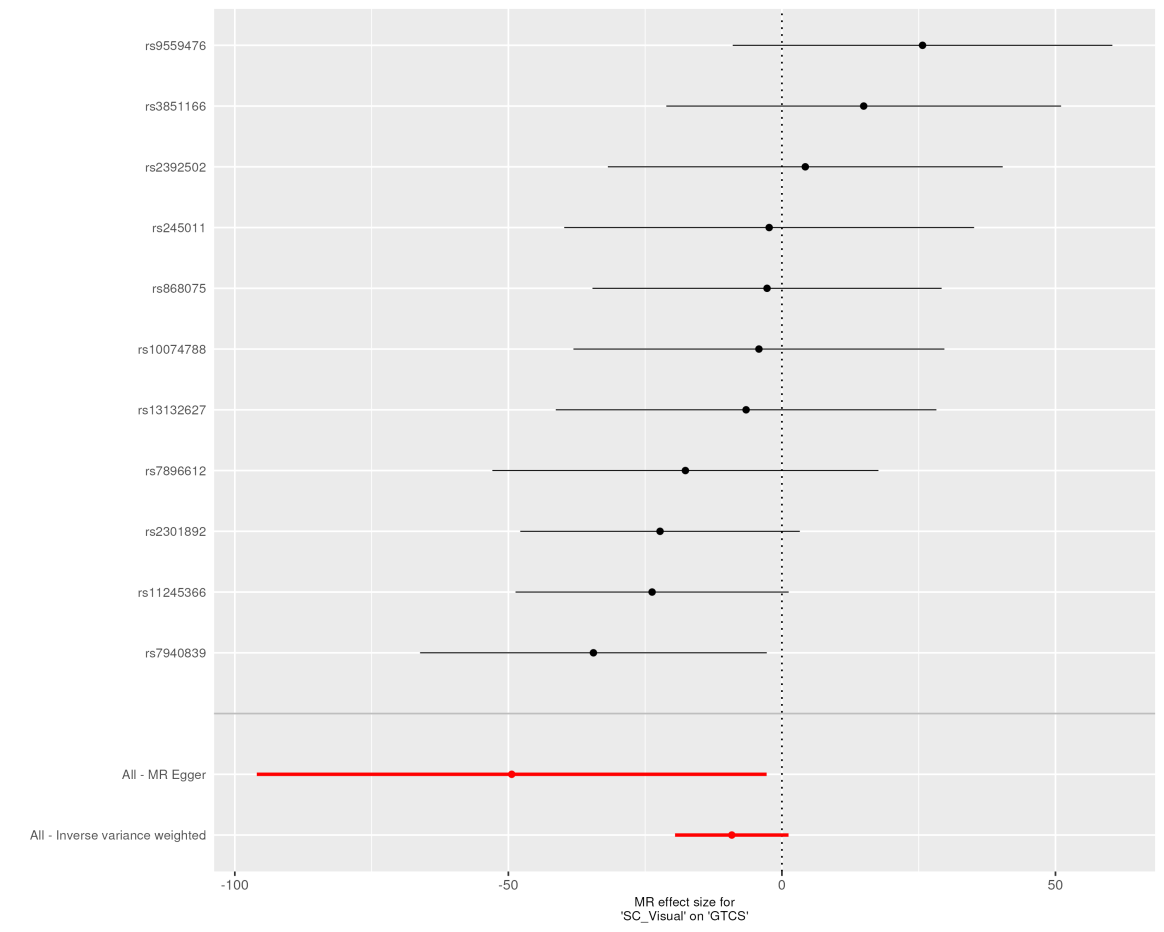


**Supplementary Figure 10** The funnel plots of the association between genetic predicted structural connectivity on epilepsy and subtypes in MR analysis.

Supplementary Figure 10 (a). The funnel plots of the association between genetic predicted frontoparietal on CAE in MR analysis.


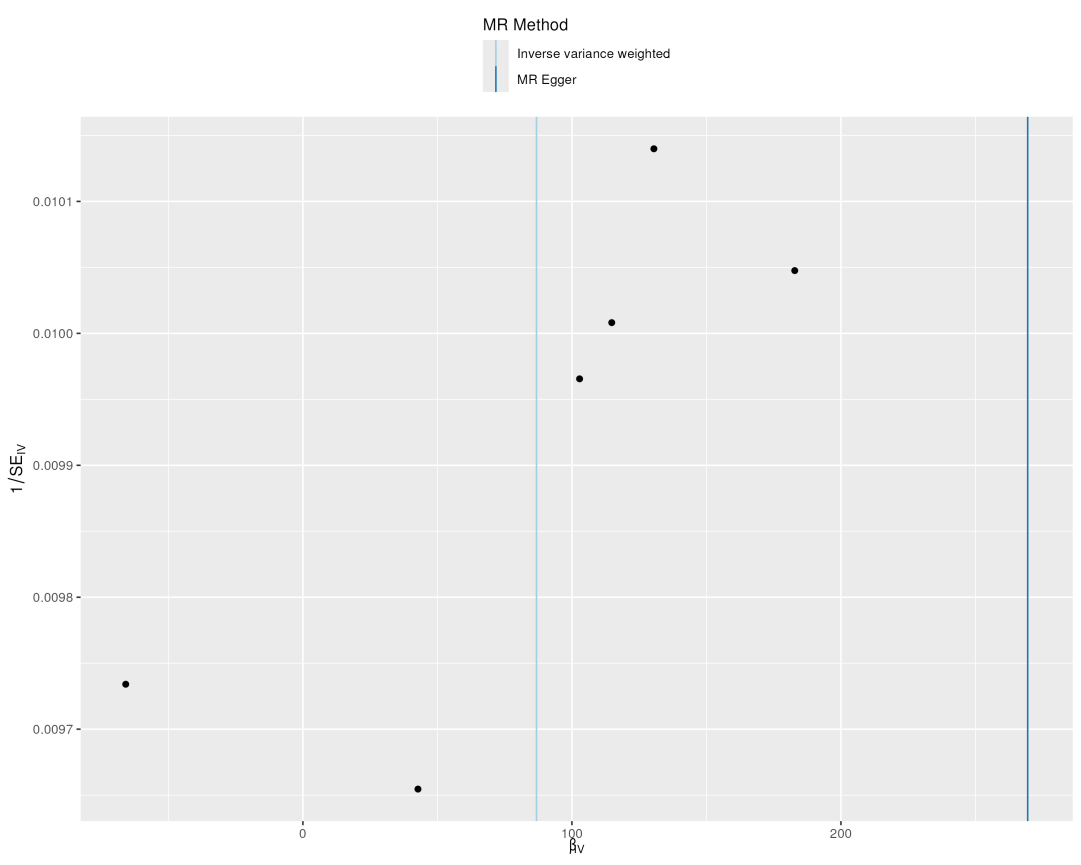


Supplementary Figure 10 (b). The funnel plots of the association between genetic predicted global on all epilepsy in MR analysis.


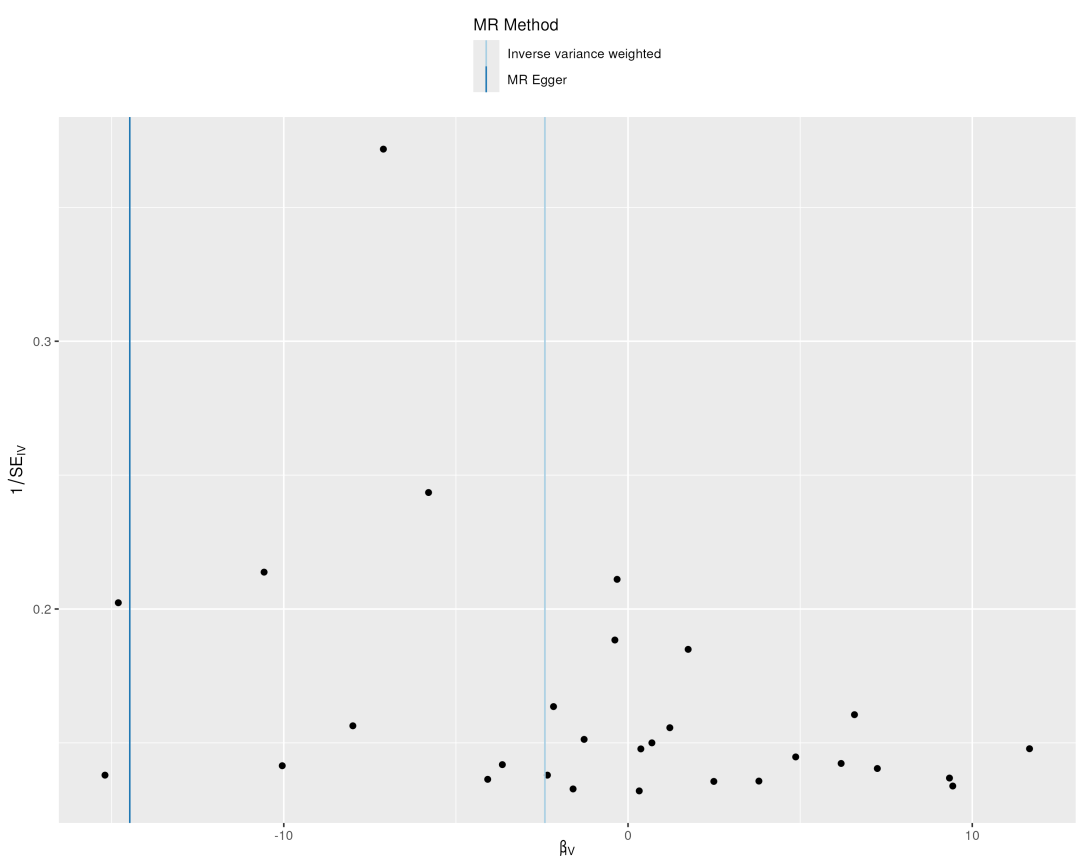


Supplementary Figure 10 (c). The funnel plots of the association between genetic predicted visual on GTCS in MR analysis.


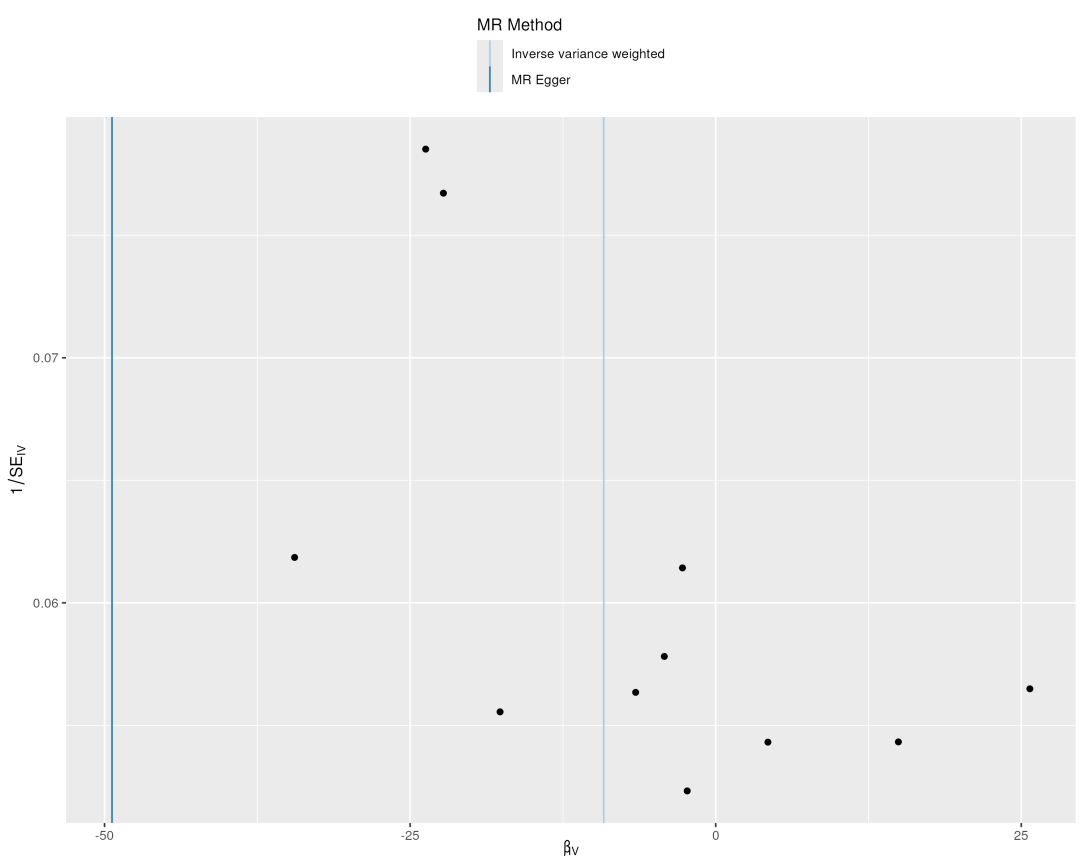


**Supplementary Figure 11** The leave-one-out analysis of the association between genetic predicted structural connectivity on epilepsy and subtypes in MR analysis.

Supplementary Figure 11 (a). The leave-one-out analysis of the association between genetic predicted frontoparietal on CAE in MR analysis.


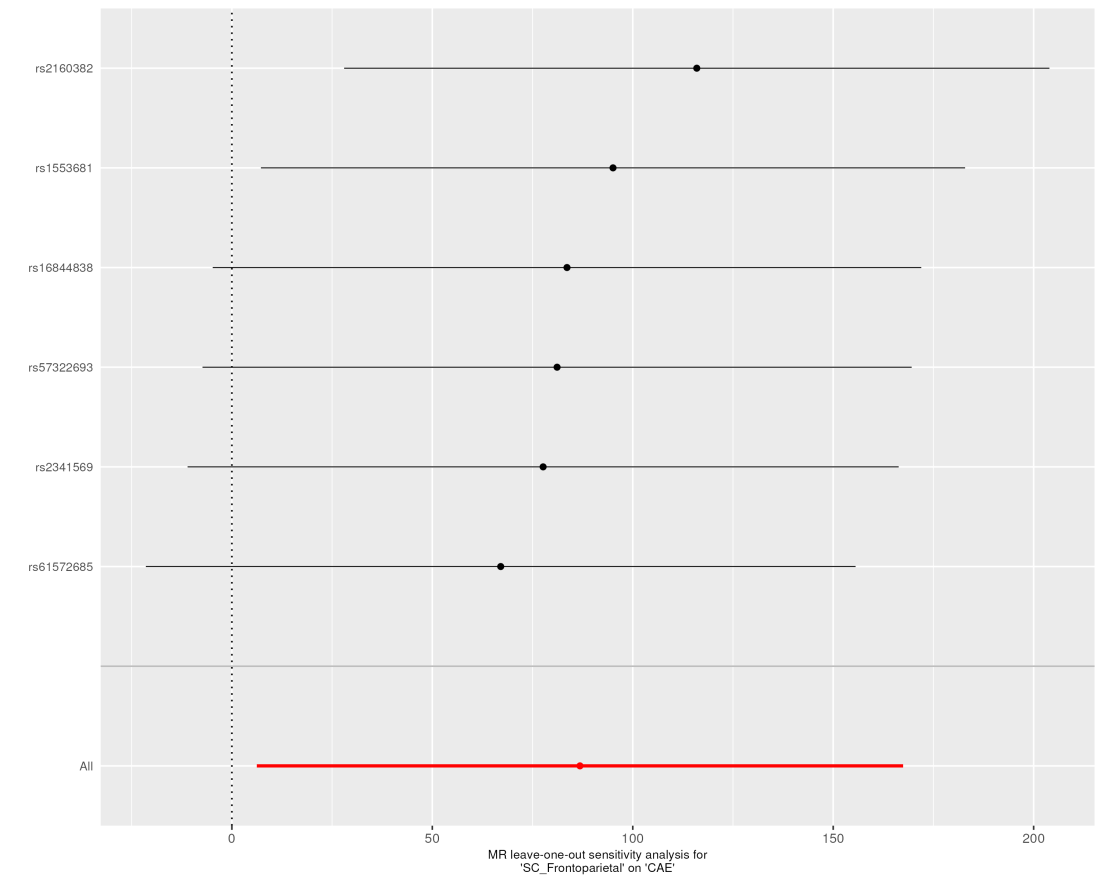


Supplementary Figure 11 (b). The leave-one-out analysis of the association between genetic predicted global on all epilepsy in MR analysis.


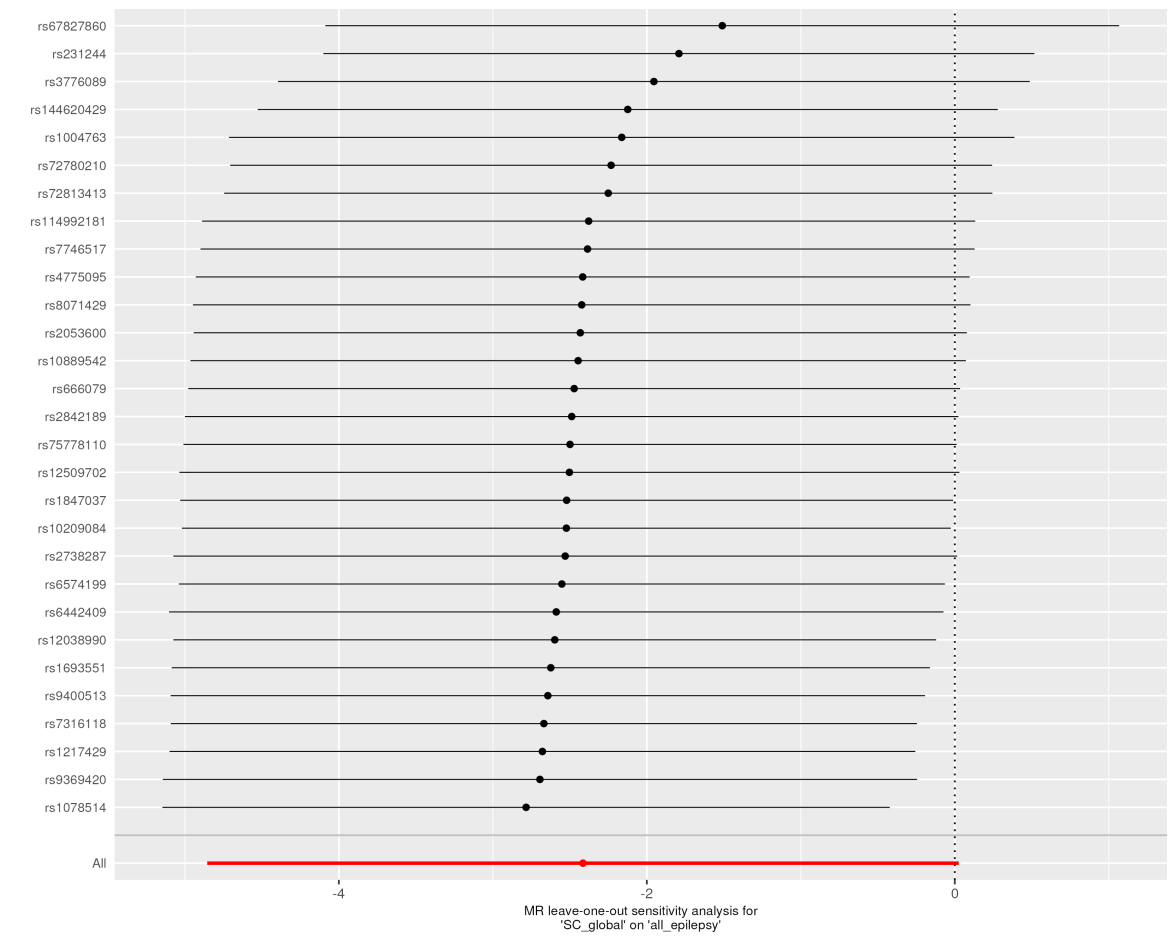


Supplementary Figure 11 (c). The leave-one-out analysis of the association between genetic predicted visual on GTCS in MR analysis.


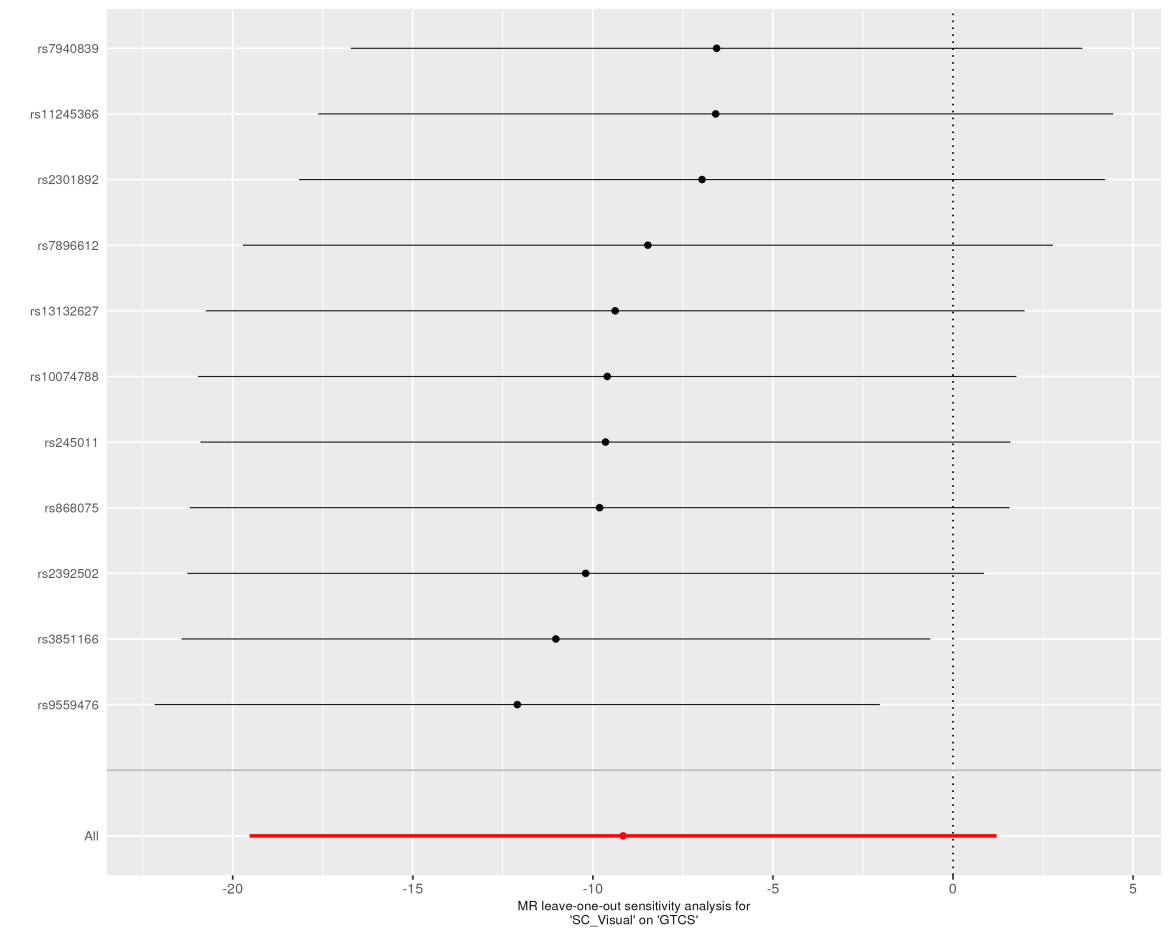


**Supplementary Figure 12** The scatter plots of the association between genetic predicted structural connectivity on epilepsy and subtypes in MR analysis.

Supplementary Figure 12 (a). The scatter plots of the association between genetic predicted frontoparietal on CAE in MR analysis.


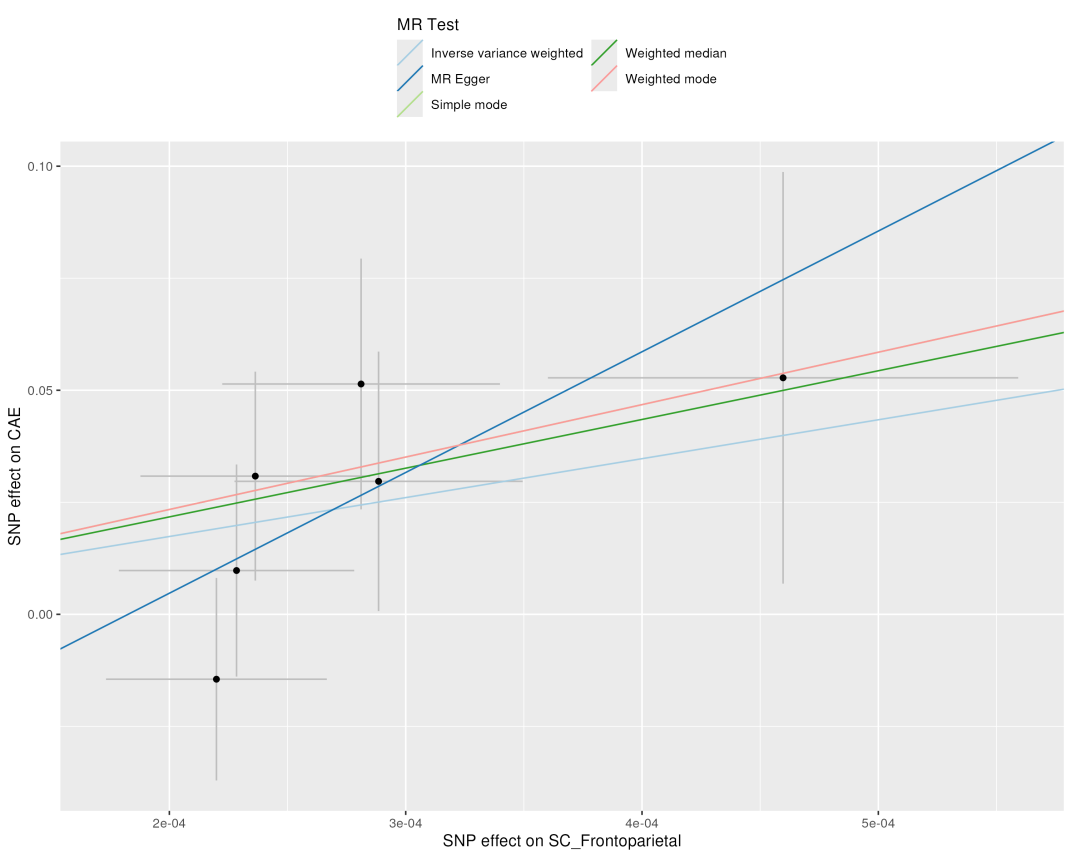


Supplementary Figure 12 (b). The scatter plots of the association between genetic predicted global on all epilepsy in MR analysis.


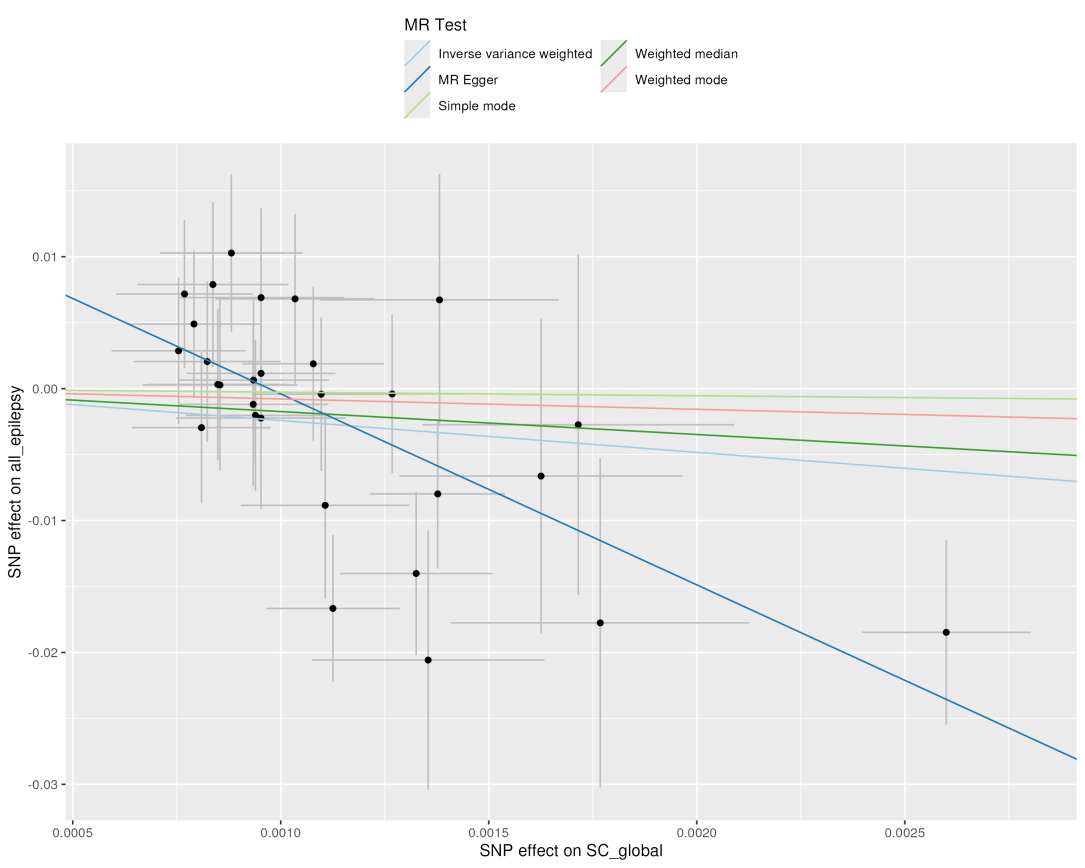


Supplementary Figure 12 (c). The scatter plots of the association between genetic predicted visual on GTCS in MR analysis.


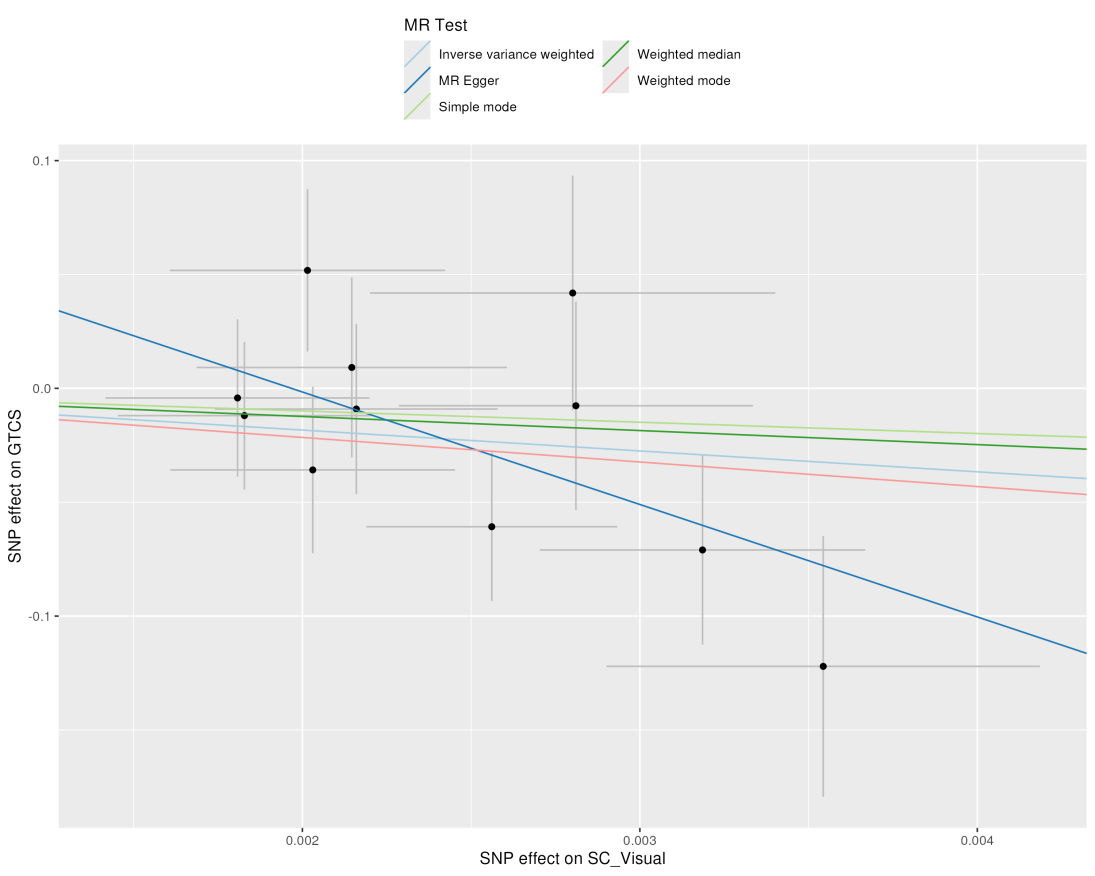

Supplement: Supplementary 1 — Tables S1 to S11 Figs. S1 to S12 [file hds.0283.f1.zip › Supplementary Information_Supplementary tables and figures.docx]
